# Global folate status in women of reproductive age: a systematic review with emphasis on methodological issues

**DOI:** 10.1111/nyas.13963

**Published:** 2018-09-21

**Authors:** Lisa M. Rogers, Amy M. Cordero, Christine M. Pfeiffer, Dorothy B. Hausman, Becky L. Tsang, Luz María De‐Regil, Jorge Rosenthal, Hilda Razzaghi, Eugene C. Wong, Aliki P. Weakland, Lynn B. Bailey

**Affiliations:** ^1^ Evidence and Programme Guidance, Department of Nutrition for Health and Development World Health Organization Geneva Switzerland; ^2^ National Center on Birth Defects and Developmental Disabilities Centers for Disease Control and Prevention Atlanta Georgia; ^3^ National Center for Environmental Health Centers for Disease Control and Prevention Atlanta Georgia; ^4^ Foods and Nutrition Department University of Georgia Athens Georgia; ^5^ Food Fortification Initiative Atlanta Georgia; ^6^ Global Technical Services Nutrition International Ottawa Ontario Canada; ^7^ Oak Ridge Institute for Science and Education Oak Ridge Tennessee; ^8^ Core Engagement LLC Fort Collins Colorado

**Keywords:** global, folate, status, women, methodological issues

## Abstract

Inadequate folate status in women of reproductive age (WRA) can lead to adverse health consequences of public health significance, such as megaloblastic anemia (folate deficiency) and an increased risk of neural tube defect (NTD)‐affected pregnancies (folate insufficiency). Our review aims to evaluate current data on folate status of WRA. We queried eight databases and the World Health Organization Micronutrients Database, identifying 45 relevant surveys conducted between 2000 and 2014 in 39 countries. Several types of folate assays were used in the analysis of blood folate, and many surveys used folate cutoffs not matched to the assay. To allow better comparisons across surveys, we attempted to account for these differences. The prevalence of folate deficiency was >20% in many countries with lower income economies but was typically <5% in countries with higher income economies. Only 11 surveys reported the prevalence of folate insufficiency, which was >40% in most countries. Overall, folate status data for WRA globally are limited and must be carefully interpreted due to methodological issues. Future surveys would benefit from using the microbiologic assay to assess folate status, along with assay‐matched cutoffs to improve monitoring and evaluation of folic acid interventions, thus informing global efforts to prevent NTDs.

## Introduction

Folate is essential for DNA replication and normal cell formation and growth. Folate deficiency and insufficiency can result in adverse health outcomes. Folate insufficiency in women of reproductive age (WRA) can lead to pregnancies affected by neural tube defects (NTDs).^1^ NTDs, such as spina bifida and anencephaly, affect the development of the brain and spine, can lead to an early death or lifelong disability, and are among the most serious and most common congenital anomalies.^2^ Most NTDs are preventable if women consume enough folic acid, a synthetic form of folate, prior to and during early pregnancy.^3^, ^4^ NTDs are among the most important congenital burdens in the neonatal period and it is estimated that annually 260,100 NTD‐affected pregnancies occur worldwide.^5^ As countries continue to make progress in reducing child mortality from infectious diseases, birth defects become a more significant cause of under‐5 mortality in many countries.^6^ As a result, in 2010, the 63rd World Health Assembly adopted a resolution calling for all member states of the WHO to promote the primary prevention of birth defects.^7^ Reducing the occurrence of NTDs will be vital for countries to achieve the United Nations Sustainable Development Goals related to health by 2030.^8^, ^9^

Folate deficiency can also lead to megaloblastic anemia in adults and children through impaired DNA synthesis and cell division, therefore leading to ineffective erythropoiesis.^10^, ^11^ Megaloblastic anemia may also be caused by vitamin B12 deficiency, and other types of anemia can be caused by deficiencies of iron or other micronutrients, infection, or inherited genetic disorders that affect hemoglobin synthesis or red blood cell (RBC) production. Anemia, from all causes, affects about one‐third of WRA worldwide.^12^ One of the World Health Organization's (WHO) Global nutrition targets is a 50% reduction in anemia in WRA by 2025 (WHO Global nutrition target 2).^13^

Many countries have sought to increase folic acid consumption through fortification of staple foods and targeted supplementation programs. To enhance these efforts, the Micronutrient Forum, with support from the Bill & Melinda Gates Foundation, convened a “Technical Consultation on Folate Status in Women and Neural Tube Defect Prevention” to develop a roadmap to better inform and prioritize investments in folate‐preventable NTDs.^14^ The consultation also sought to identify the global prevalence of folate insufficiency in WRA and determine the fraction of NTDs currently attributable to folate insufficiency. Global folate status was first reviewed in a 2005 WHO Technical Consultation on folate and vitamin B12 deficiencies.^15^ However, more recent information on folate status of WRA, particularly using the newly recommended WHO cutoff for folate insufficiency,^1^ has not been assessed.

There is a variety of assays, such as the microbiological assay (MBA), protein‐binding assay (PBA), and high‐performance liquid chromatography coupled to tandem mass spectrometry (HPLC–MS/MS), and various methods within the different types of assays, available for the assessment of serum or plasma and RBC folate concentrations.^16^ Large differences in results among these folate assays have been observed, which hinder the direct comparison of folate status data when different types of assays are used.^17^, ^18^, ^19^, ^20^ Assay‐specific cutoff values have been proposed for defining folate status and recent articles using data from the United States and Belize have shown the impact of misinterpretation when inappropriate cutoff values are applied to data not matched for the assay.^21^, ^22^

The primary objective of our review article is to identify and evaluate the current evidence on folate status of WRA. We also assess the types of folate assays and cutoff values used and highlight opportunities to improve their use in the monitoring and evaluation of folic acid interventions, thus informing global efforts to prevent anemia and NTDs.

## Methods

Details of the protocol for this systematic review were registered on PROSPERO and can be accessed at http://www.crd.york.ac.uk/PROSPERO/display_record.php?ID=CRD42017072645.

### Identification of surveys

A systematic literature search on the human surveys of blood folate concentration and status (i.e., population prevalence of folate deficiency or insufficiency) in WRA was conducted by a reference librarian for articles published between January 2000 and June 2017. The databases searched were MEDLINE (Ovid), PubMed, Embase (Ovid), Scopus, CINAHL, Global Health (Ovid), POPLINE, and the Cochrane Central Register of Controlled Trials (CENTRAL). The following search terms and their variants were used: (((national) AND (survey)) OR ((population) AND (prevalence))) AND ((folate status) OR (folate deficiency) OR (folic acid deficiency) OR (serum folate) OR (vitamin B12 deficiency) OR (RBC folate) OR (blood folate) OR (folate concentration)). Full search strategies are available in Appendix S1 (online only). There were no limitations based on language.

We scanned the reference lists of all relevant systematic reviews returned in the search results to identify any relevant surveys potentially missed by the systematic electronic search. We also searched the Micronutrients Database of the WHO's Vitamin and Mineral Nutrition Information System (VMNIS) for any additional surveys or reports not already identified.^23^ The WHO Micronutrients Database aims to systematically retrieve and summarize data on the assessment of vitamin and mineral status of populations.

### Inclusion and exclusion criteria

To be eligible for inclusion in the systematic review, surveys must have reported serum, plasma, or RBC folate concentrations for WRA (defined here as premenopausal women aged 12–49 years), representative of the national, regional (within the country), or first administrative (e.g., state, province, and canton) levels. We defined regional as a subnational area comprising more than one first administrative level within a country. Some studies were conducted only in or included adolescent girls. When data were reported for WRA disaggregated by physiological status (i.e., pregnant, lactating, and nonpregnant), we used data for nonpregnant women; however, when data were not disaggregated, we used the available data. We excluded surveys solely reporting data on pregnant or lactating WRA due to differences in folate metabolism of these population subgroups. In populations that were not limited to or did not specifically stratify by the target population group (i.e., women aged 12–49 years); at least 51% of the population had to have met the above criteria for inclusion. We also excluded surveys conducted in institutionalized populations or those selected based on a health condition as these populations are not representative of the general population.

### Selection of surveys

Two teams of reviewers (A.M.C. + H.R., J.R. + E.C.W.) each screened approximately half of all titles and abstracts retrieved in the search results against inclusion and exclusion criteria. The reviewers flagged potentially relevant records for full‐text review, reviewed the full text of selected records for eligibility, and recorded reasons for excluding ineligible records. We settled any discrepancies between reviewer teams during the title or abstract review through discussion and consultation. All full‐text records identified as relevant were then reviewed by a fifth coauthor (L.M.R.), who was independent of the search and screening process, to confirm representativeness based on the standards set for the WHO Micronutrients Database.

### Data extraction

We retrieved the original reports of all records included in this review and extracted the data most relevant to the purposes of the review from the published manuscripts or official survey reports. We entered data from all records meeting the inclusion criteria into the WHO Micronutrients Database if they had not been previously entered. The WHO Micronutrients Database uses a system of data entry plus verification (two independent individuals). We then downloaded data for the relevant key variables (e.g., prevalence of folate deficiency, sample size, and analytical methods) from the database to a spreadsheet. We reviewed these data for accuracy and extracted supplemental data (blood sampling, processing, transport, and storage) from the records.

### Blood folate cutoffs for deficiency and insufficiency

The WHO^24^ and the Institute of Medicine (IOM)^25^ have recommended cutoffs for folate deficiency based on the use of blood folate as a hematological indicator of megaloblastic anemia or metabolic indicator of rising homocysteine concentrations. It is generally considered that plasma or serum folate reflect recent folate intake and are likely indicative of the dietary intake of the populations. Serum or plasma folate concentrations less than 7 nmol/L indicate negative folate balance, an early stage of folate deficiency (Table 1). If negative folate balance persists (weeks or months), then megaloblastic changes in the blood and bone marrow become apparent, increasing the risk of megaloblastic anemia.^10^, ^24^ However, an even earlier stage of folate deficiency is expressed by rising homocysteine concentrations (a metabolic indicator), which appear at higher levels of serum or plasma folate (10 or 14 nmol/L).^24^, ^26^

**Table 1:** Red blood cell and serum/plasma folate concentrations for defining folate status

	Cutoff value (nmol/L)^a^			
Interpretation	Traditional MBA (folic acid calibrator)^b^	Contemporary MBA (folic acid calibrator)^c^	Contemporary MBA (5‐methyl‐THF calibrator)^c^^,^ d	Bio‐Rad RIA^e^
**Red blood cell folate**				
Insufficiency, increased risk of NTD		<906^1^, ^24^	<748^21^	
Deficiency defined based on rising homocysteine concentration as a metabolic indicator			<624^21^	<340^24^
Deficiency defined based on risk of megaloblastic anemia signified by appearance of hypersegmented neutrophils	<305^25^, ^27^		<305^21^	<215^21^
**Serum/plasma folate**				
Deficiency defined based on rising homocysteine concentration as a metabolic indicator			<14^21^	<10^24^
Deficiency defined based on risk of megaloblastic anemia (negative folate balance)	<7^10^, ^24^		<7^21^	<5^21^

a Values are rounded to the nearest integer. Blank cells indicate that no cutoff value has been established.

b MBA with wild‐type microorganism.

c MBA with chloramphenicol‐resistant strain.

d Equivalent to the CDC MBA method.

e The Bio‐Rad RIA was used for many years in the U.S. NHANES. Folate data from NHANES III were derived using this assay and used in developing the cutoff values <10 nmol/L for serum folate and <340 nmol/L for red blood cell folate.

5‐methyl‐THF, 5‐methyl‐tetrahydrofolate; CDC, the Centers for Disease Control and Prevention; MBA, microbiologic assay; NHANES, National Health and Nutrition Examination Survey; NTDs, neural tube defects; RIA, radioimmunoassay.

Disclaimer: The mention of specific companies or certain manufacturers’ products does not imply that they are endorsed or recommended by the WHO in preference to others of a similar nature that are not mentioned. Errors and omissions excepted, the names of proprietary products are distinguished by initial capital letters.

RBC folate reflects longer‐term folate status (preceding 120 days, i.e., the average life span of red cells) and would not reflect any short‐term, transient dietary folate insufficiencies reflected in serum/plasma folate.^16^ Megaloblastic changes (appearance of hypersegmented neutrophils and the early stage of megaloblastic anemia) tend to appear with RBC folate concentrations <305 nmol/L^25^, ^27^ (Table 1). Thus, folate deficiency prevalence estimates in the literature can vary based on the type of specimen (plasma/serum versus RBC), type of indicator (rising homocysteine versus megaloblastic changes), and type of assay (see below).

In 2015, WHO published the guideline on an RBC folate cutoff of <906 nmol/L for assessing folate insufficiency, representing the risk of NTDs for WRA at the population level.^1^ This value can also be useful in the assessment of the need for and effectiveness of folic acid interventions designed to prevent NTDs.^28^ No threshold for serum folate could be established as an indicator of NTD risk reduction due to insufficient data.^1^

To simplify the current work, we focused on folate insufficiency defined as the risk of NTDs and on folate deficiency defined as the risk of megaloblastic anemia in consideration of the current priorities in public health for the prevention of NTDs and anemia.

### Critical assessment of blood collection and analysis methods and reporting of blood folate data

#### Assessment of blood specimen handling and storage

From each record, we extracted information about how the blood was collected, transported, processed, stored, and analyzed. Data were extracted by one of four authors (D.B.H., L.B.B., L.M.R., and C.M.P.) and evaluated by one author (C.M.P.).

#### Assessment of possible bias in folate concentrations and prevalence estimates due to the choice of assay and cutoff(s)

The surveys included in the review measured serum, plasma, and/or RBC folate concentrations using a variety of assay types and methods (Appendix S2, online only). Because of the large differences in results among these different types of folate assays, and because assay‐specific cutoff values may be needed to define folate status (e.g., deficiency and insufficiency),^17^, ^18^ we aimed to systematically consider these differences in this review.

The WHO recommends using the MBA to obtain comparable results for blood folate concentrations across countries.^1^ However, even when using the MBA, use of different folate calibrators and microorganism preparations may lead to different results and may require cutoff adjustments to make comparisons across MBA methods.^20^ For example, the Centers for Disease Control and Prevention (CDC) contemporary MBA (MBA_C_) calibrated with 5‐methyltetrahydrofolate (5‐methyl‐THF) generates lower results than the MBA_C_ with folic acid calibration, requiring a lower cutoff of <748 instead of <906 nmol/L RBC folate to indicate folate insufficiency.^21^ It is not known how today's MBA_c_ with 5‐methyl‐THF calibration compares to the traditional MBA (MBA_T_) with folic acid calibration, which was used decades ago to establish the deficiency cutoffs of <305 nmol/L for RBC folate and <7 nmol/L for serum folate.^24^, ^25^, ^27^, ^29^ Indirect evidence suggests that these two assays likely produce similar results.^21^

To allow better comparisons across surveys, we tried to account for assay differences by using data from two proficiency testing programs to compare the performance of the assay used in any given survey with the performance of a single comparison assay. For reasons detailed in Appendix S2 (online only), we designated the CDC MBA_C_ calibrated with 5‐methyl‐THF as the comparison assay. In the proficiency testing programs, each participating laboratory runs its assay on the same proficiency panel. Using that information, we estimated an “assay factor,” or the extent to which one assay tests higher or lower than another, by calculating the ratio of the survey assay's results to the CDC MBA_C_ results (details are described in Appendix S2, online only).

We also estimated a “cutoff factor,” calculated as the ratio of the survey cutoff to the MBA_C_ cutoff. By estimating how the various assays performed relative to the MBA_C_ using the assay factor, and by considering the cutoff values used in each survey for defining folate deficiency or insufficiency using the cutoff factor, we estimated a “prevalence factor” (assay factor divided by cutoff factor) to better assess whether the reported prevalence estimates are likely correct (prevalence factor >0.85 and <1.15) or may represent an under‐ (prevalence factor ≥1.15) or overestimation (prevalence factor ≤0.85).

## Results

### Survey selection

**Figure 1:**
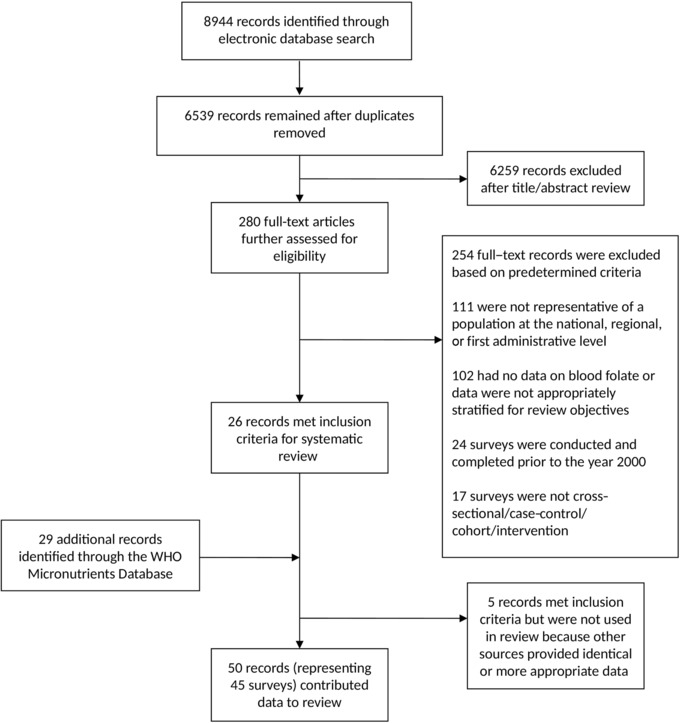
Study flow diagram.

The electronic search yielded a total of 8944 records across eight databases (Fig. 1). After the removal of duplicates, 6539 unique records remained. Following title and abstract screening, 280 records were identified as potentially eligible and assessed for eligibility with the full‐text review. Of these, 26 records were eligible for inclusion in the review. While excluded records could have failed to meet multiple inclusion criteria, the most common reason noted by the reviewers (*n* = 111) was the lack of representativeness of the population at the first administrative level or above.

We identified an additional 29 records through the WHO Micronutrients Database. Following a thorough assessment of all 55 records meeting the inclusion criteria, we excluded five records because identical data or similar data with more appropriate presentation (e.g., more appropriately stratified by target population) were available from other included records. In total, 45 surveys from 50 records were included in our review.

The sample size of each survey is shown in Supplementary Figure S1 (online only), stratified by serum/plasma and RBC folate (where applicable) and classified by gross national income, based on the most recent World Bank classification.^30^

### Critical assessment of blood collection and analysis method and reporting of blood folate data

#### Assessment of blood specimen handling and storage

Most surveys reported sufficient information about specimen handling and storage to allow an assessment of the appropriateness of these procedures based on established criteria.^16^ In most cases, appropriate procedures were followed to ensure specimen integrity (Supplementary Table S1, online only). In a few cases, storage or handling of samples under suboptimal conditions may have led to folate losses (Côte d'Ivoire 2007,^31^ Sierra Leone 2013,^32^, ^33^ the United Kingdom 2000–2001,^34^ and 2008–2012,^35^ and Uzbekistan 2008^36^); in other cases, incomplete information was provided (Afghanistan 2013,^37^ Australia 2011–2012,^38^ Cambodia 2014,^39^ Ecuador 2012,^40^ Jordan 2010,^41^, ^42^ Lebanon 2003,^43^ Mongolia 2004,^44^ and Sweden 2010–2011^45^). No information was reported in one survey (Newfoundland, Canada^46^).

#### Assessment of possible bias in folate concentrations and prevalence estimates due to the choice of assay and cutoff(s)

Of the 45 surveys included in this review, 27 (60%) used one of several types of PBA to assess folate status, 13 (29%) used MBA, and two used both HPLC–MS/MS and MBA (Supplementary Table S1, online only). Three did not report the type of assay used.

**Figure 2:**
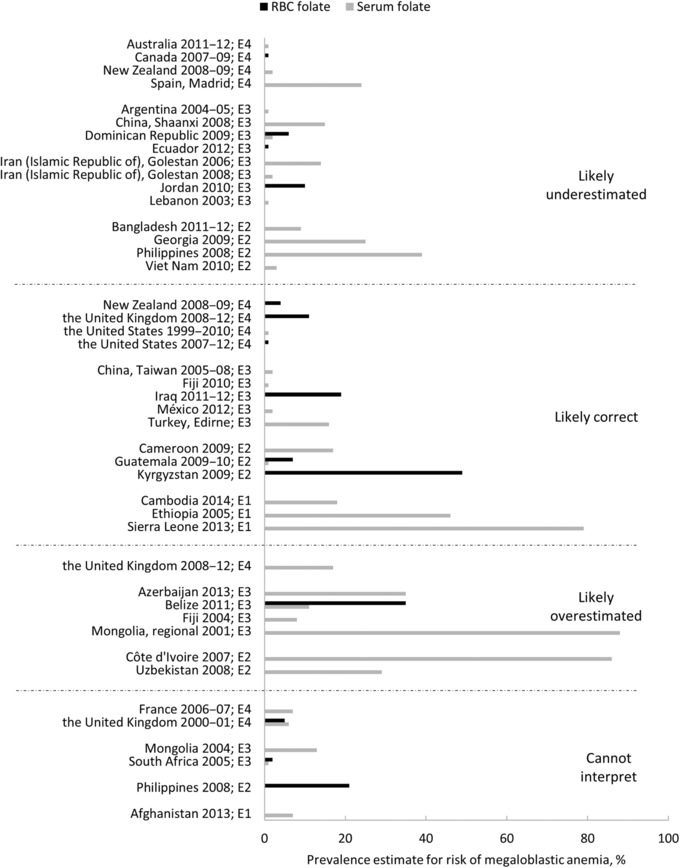
The reported prevalence of folate deficiency in women of reproductive age by the survey, indicating an interpretation of the prevalence estimate based on the assay and cutoff used in the survey. An assay factor, or the extent to which one assay measures higher or lower than another, was calculated as the ratio of the survey assay's results to the CDC MBA_C_. Similarly, a cutoff factor was calculated as the ratio of the survey cutoff to the MBA_C_ cutoff. A prevalence factor (assay factor divided by cutoff factor) was calculated to estimate whether the reported prevalence estimates are likely correct (prevalence factor >0.85 and <1.15) or may represent an under‐ (prevalence factor ≥1.15) or overestimation (prevalence factor ≤0.85). Gray bars indicate serum/plasma folate and black bars indicate RBC folate. Prevalence estimates reported as <1% (or 0%) are shown as 1%. For two surveys (New Zealand 2008–2009^75^ and the Philippines 2008^51^), the interpretation of the reported prevalence was different for serum and RBC folate; thus, results by sample type are listed separately in the appropriate category. Surveys that did not report a prevalence for folate deficiency (*n* = 5) are not shown. CDC, the Centers for Disease Control and Prevention; MBA_C_, contemporary microbiologic assay. The abbreviation after each survey indicates the economy: E1, low‐income; E2, lower‐middle‐income; E3, upper‐middle‐income; E4, high‐income.

**Figure 3:**
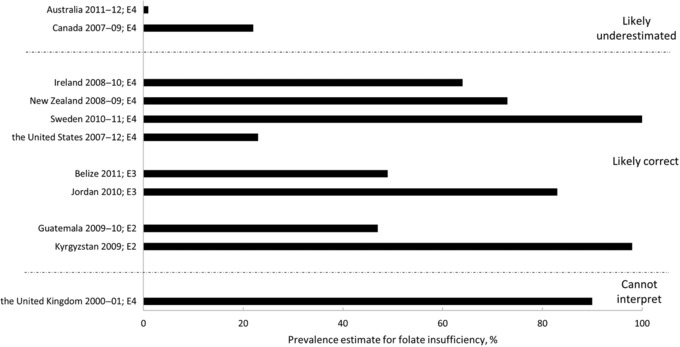
The reported prevalence of folate insufficiency, based on red blood cell folate concentrations, in women of reproductive age by the survey, indicating an interpretation of the prevalence estimate based on the assay and cutoff used in the survey. An assay factor, or the extent to which one assay measures higher or lower than another, was calculated as the ratio of the survey assay's results to the CDC MBA_C_. Similarly, a cutoff factor was calculated as the ratio of the survey cutoff to the MBA_C_ cutoff. A prevalence factor (assay factor divided by cutoff factor) was calculated to estimate whether the reported prevalence estimates are likely correct (prevalence factor >0.85 and <1.15) or may represent an under‐ (prevalence factor ≥1.15) or overestimation (prevalence factor ≤0.85). Prevalence estimates reported as <1% (or 0%) are shown as 1%. Surveys that did not report a prevalence of folate insufficiency (*n* = 34) are not shown. CDC, the Centers for Disease Control and Prevention; MBA_C_, contemporary microbiologic assay. The abbreviation after each survey indicates the economy: E1, low‐income; E2, lower‐middle‐income; E3, upper‐middle‐income; E4, high‐income.

We aimed to report folate status in WRA based on cutoffs recommended by the WHO^24^ and the IOM,^25^ focusing on folate insufficiency and on deficiency defined by the risk of megaloblastic anemia, with a few modifications and additions to account for assay differences (Table 1 and Appendix S2, online only). Not all survey data were reported using these cutoffs, and the actual cutoffs reported are noted in Tables 2, 3, 4, 5. The reported mean serum and RBC folate concentrations and the suitability of the mean concentration based on the survey's assay are summarized in Supplementary Figures S2 and S3 (online only). The prevalence of folate deficiency and insufficiency for WRA and the suitability of the prevalence estimate based on both the survey's assay and cutoff used in the survey, using the factor values defined below, is summarized in Figures 2 and 3, respectively. In the following, we report on the survey findings relative to the prevalence factor. For detailed information on how the survey assay was performed, the reader is referred to Supplementary Table S1 (online only).

**Table 2:** Folate status of women of reproductive age based on serum/plasma folate samples collected in representative national surveys^a^ in countries with low‐income economies

Country (year)	Sample size	Age range (years)	Fasting status	Survey assay	Assay factor^b^	Serum folate^c^ nmol/L (95% CI)	Prevalence of folate deficiency (95% CI) and survey cutoff^d^ in nmol/L	Cutoff factor^e^	Prevalence factor^f^	Comments regarding the prevalence of folate deficiency^g^
Afghanistan (2013)^37^	741	10–19	Not reported	Not reported	NA	Not reported	7% at <7	1.0	NA	Cannot interpret (survey assay not known)
Cambodia (2014)^39^	725	15–49	Casual	PBA (IA; Roche cobas® e411)	1.59	14.1#	18% at <10	1.43	1.11	Likely correct
Ethiopia (2005)^49^	970	15–49	Fasting	PBA (IA; Roche Elecsys®)	1.15	12.6	46% at ≤9	1.29	0.89	Likely correct
Sierra Leone (2013)^32^, ^33^	766	15–49	Casual	PBA (IA; Roche cobas® e411)	1.59	8.6 (8.1–9.2)	79% (74–84) at <10	1.43	1.11	Likely correct

a All surveys are nationally representative household‐based, cross‐sectional surveys, except for the survey from Ethiopia which is a cross‐sectional, community‐based survey.

b Ratio of survey assay results to MBA_C_ results. Calculated from proficiency testing data generated as close as possible to the time of the survey as the ratio between survey assay results and results obtained by the contemporary microbiologic assay calibrated with 5‐methyl‐THF and conducted at the Centers for Disease Control and Prevention.

c Values represent mean folate concentrations except as indicated: ^#^median.

d Cutoff values represent those indicated in the corresponding survey report or publication; prevalence and cutoff values have been rounded to the nearest whole integer.

e Ratio of survey folate deficiency cutoff to MBA_c_ folate deficiency cutoff (7 nmol/L for serum folate).

f Assay factor divided by cutoff factor.

g Prevalence estimates were considered likely correct if the calculated prevalence factor was >0.85 and <1.15, underestimated if the prevalence factor was ≥1.15, or overestimated in the prevalence factor was ≤0.85.

note: Note that there were no surveys in countries with low‐income economies that assessed status based on red blood cell folate concentrations.

IA, immunoassay; MBA_C_, contemporary microbiological assay; PBA, protein binding assay; NA, not applicable.

Disclaimer: The mention of specific companies or certain manufacturers’ products does not imply that they are endorsed or recommended by the WHO in preference to others of a similar nature that are not mentioned. Errors and omissions excepted, the names of proprietary products are distinguished by initial capital letters.

**Table 3:** Folate status of women of reproductive age based on RBC or serum/plasma folate samples collected in representative national surveys^a^ in countries with lower‐middle‐income economies

Country (year)	Sample size	Age range (years)	Fasting status	Survey assay	Assay factor^b^	Folate^c^ nmol/L (95% CI)	Prevalence of folate insufficiency (95% CI) and survey cutoff^d^ in nmol/L	Prevalence of folate deficiency (95% CI) and survey cutoff^d^ in nmol/L	Cutoff factor^e^	Prevalence factor^f^	Comments regarding the prevalence of folate deficiency^g^
**Assessment based on RBC folate concentration**											
Guatemala (2009–2010)^47^, ^50^, ^83^	1448	15–49	Casual	MBA_C_ (5‐methyl‐THF calibrator)	1.00	725ǂ (711–738) 766#	47% (43–51) at <748	7% at <317 [9% at <340]	1.03	0.97	Likely correct
Kyrgyzstan (2009)^48^	735	≥17^h^	Not reported	MBA_C_ (dried blood spots; folic acid calibrator)	1.20	Not reported	98% (96–99) at <906	49% (42–57) at <342	1.12	1.07	Likely correct
The Philippines (2008)^51^	2119	13–45	Fasting	PBA (RIA; DPC Dual Count™ Solid Phase No Boil)	NA	592ǂ (568–618)	Not reported	21% (19–23) at <397	1.30	NA	Cannot interpret (ratio of survey assay to the CDC MBA not known)
**Assessment based on serum/plasma folate concentration**											
Bangladesh (2011–2012)^52^	849	15–49	Casual	PBA (IA; Roche cobas® e601)	1.63	Not reported	NA	9% (5–13) at <7	1.00	1.63	Likely underestimated
Cameroon (2009)^53^	390	15–49	Casual	PBA (RIA; MP Biomedicals SimulTRAC‐SNB)	1.34	18#	NA	17% (11–23) at <10	1.43	0.94	Likely correct
Côte d'Ivoire (2007)^31^	853	15–49	Not reported	MBA_C_ (folic acid calibrator)	1.20	5.9	NA	86% at <10	1.43	0.84	Likely overestimated
Georgia (2009)^54^	407	15–49	Not reported	MBA_C_ (folic acid calibrator)	1.20	16.3 (14.3–18.6)	NA	25% at <7 [37% at <9]	1.00	1.20	Likely underestimated
Guatemala (2009–2010)^50^, ^83^	1448	15–49	Casual	MBA_C_ (5‐methyl‐THF calibrator)	1.00	30ǂ (29–31)	NA	<1% at <7 [5% at <10]	1.00	1.00	Likely correct
The Philippines (2008)^51^	2119	13–45	Fasting	PBA (RIA; DPC Dual Count™ Solid Phase No Boil)	1.16	7.4ǂ (7.1–7.7)	NA	39% (36–42) at <7	1.00	1.16	Likely underestimated
Uzbekistan (2008)^36^	2563	15–49	Not reported	MBA_C_ (5‐methyl‐THF calibrator)	1.00	11.9ǂ (11.7–12.2)	NA	29% (27–31) at <10	1.43	0.70	Likely overestimated
Vietnam (2010)^55^	1472	15–49	Casual	MBA_C_ (folic acid calibrator)	1.20	17.6#	NA	3% at <7 [25% at 7–14]	1.00	1.20	Likely underestimated

a All surveys listed are household‐based, cross‐sectional surveys representative at the national level.

b Ratio of survey assay results to MBA_C_ results. Calculated from proficiency testing data generated as close as possible to the time of the survey as the ratio between survey assay results and results obtained by the contemporary microbiologic assay calibrated with 5‐methyl‐THF and conducted at the Centers for Disease Control and Prevention.

c Values represent mean folate concentrations except as indicated: ^ǂ^geometric mean; ^#^median.

d Cutoff values represent those indicated in the corresponding survey report or publication; prevalence reported for a secondary cutoff is shown in square brackets; prevalence and cutoff values have been rounded to the nearest whole integer.

e Ratio of survey folate deficiency cutoff to MBA_C_ folate deficiency cutoff (7 nmol/L for serum folate and 305 nmol/L for RBC folate).

f Assay factor divided by cutoff factor.

g Prevalence estimates were considered likely correct if the calculated prevalence factor was >0.85 and <1.15, underestimated if the prevalence factor was ≥1.15, or overestimated if the prevalence factor was ≤0.85.

h Survey methodology for the Kyrgyzstan 2009 survey indicates that women were at least 17 years of age and were mothers of children aged 6–59 months.^48^

5‐methyl‐THF, 5‐methyltetrahydrofolate; IA, immunoassay; MBA_C_, contemporary microbiological assay; PBA, protein binding assay; RBC, red blood cell; RIA, radioimmunoassay; NA, not applicable.

Disclaimer: The mention of specific companies or certain manufacturers’ products does not imply that they are endorsed or recommended by the WHO in preference to others of a similar nature that are not mentioned. Errors and omissions excepted, the names of proprietary products are distinguished by initial capital letters.

**Table 4:** Folate status of women of reproductive age based on RBC or serum/plasma folate samples collected in representative surveys^a^ in countries with upper‐middle‐income economies

Country (year)	Sample size	Age range (years)	Fasting status	Survey assay	Assay factor^b^	Folate^c^ nmol/L (95% CI)	Prevalence of folate insufficiency (95% CI) and survey cutoff^d^ in nmol/L	Prevalence of folate deficiency (95% CI) and survey cutoff^d^ in nmol/L	Cutoff factor^e^	Prevalence factor^f^	Comments regarding the prevalence of folate deficiency^g^
**Assessment based on RBC folate concentration**											
Belize (2011)^22^	937	15–49	Casual	MBA_C_ (5‐methyl‐THF calibrator)	1.00	719ǂ (689–750)	49% at <748	35% at <624	2.05	0.49	Likely overestimated
Dominican Republic (2009)^62^	501	15–49	Not reported	MBA_C_ (folic acid calibrator)	1.20	Not reported	Not reported	6% at <317 [7% at <342]	1.04	1.15	Likely underestimated
Ecuador (2012)^40^	9042	12–49	Fasting	PBA (IA; Siemens IMMULITE® 2000)	1.62	974 886#	Not reported	<1% at <342	1.12	1.44	Likely underestimated
Iraq (2011–2012)^63^	1188	15–49	Not reported	MBA_C_ (5‐methyl‐THF calibrator)	1.00	528 (515–541)	Not reported	19% (17–21) at <342	1.12	0.89	Likely correct
Jordan (2010)^41^, ^42^	393	15–49	Not reported	MBA_C_ (folic acid calibrator)	1.20	658ǂ (612–703)	83% (78–87) at <906	10% at <317 [14% at <342]	1.04	1.15	Likely underestimated
South Africa (2005)^64^	1869	16–35	Not reported	PBA (IA; Beckman Coulter® Access® and Bayer ADVIA Centaur®)	1.79 and 1.02	2529 (2345–2710)	Not reported	2% at <616 [10% at <843]	2.02	0.89 and 0.50	Cannot interpret (2 survey assays compare differently to MBA_C_)
**Assessment based on serum/plasma folate concentration**											
Azerbaijan (2013)^65^	2584	15–49	Casual	MBA_C_ (5‐methyl‐THF calibrator)	1.00	11	NA	35% (31–39) at <10	1.43	0.70	Likely overestimated
Belize (2011)^22^	937	15–49	Casual	MBA_C_ (5‐methyl‐THF calibrator)	1.00	28ǂ (26–29)	NA	11% (9–14) at <14	2.00	0.50	Likely overestimated
China, Shaanxi (2008)^56^	1170	10–49	Not reported	PBA (RIA; MP Biomedicals SimulTRAC‐SNB)	1.34	10.4#	NA	15% at <7 [59% at 7–14]	1.00	1.34	Likely underestimated
China, Taiwan (2005–2008)^66^	261	19–44	Not reported	PBA (IA; DPC IMMULITE® 2000)	1.11	21.2	NA	2% at <7 [22% at 7–14]	1.00	1.11	Likely correct
Dominican Republic (2009)^62^	451	15–49	Not reported	MBA_C_ (folic acid calibrator)	1.20	Not reported	NA	2% at <7 [3% at <9]	1.00	1.20	Likely underestimated
Ecuador (2012)^40^	9042	12–49	Fasting	PBA (IA; Siemens IMMULITE® 2000)	1.03	37.1 34.1#	NA	Not reported^h^	NA	NA	Cannot interpret (deficiency prevalence not reported)
Fiji^i^ (2004)^60^, ^61^	738	15–44	Casual	PBA (IA; Roche E170)	1.15	18 (17.6–18.6)	NA	8% at <10	1.43	0.81	Likely overestimated
Fiji^i^ (2010)^61^	869	15–45	Not reported	PBA (IA; Roche E170)	1.42	26.6		1% at <10	1.43	0.99	Likely correct
Iran (Islamic Republic of), Golestan^j^ (2006)^58^	572	15–49	Fasting	PBA (RIA; MP Biomedicals SimulTRAC‐SNB)	1.34	13.6 (12.8–14.4)	NA	14% at <7	1.00	1.34	Likely underestimated
Iran (Islamic Republic of), Golestan^j^ (2008)^59^	600	15–49	Fasting	PBA (RIA; MP Biomedicals SimulTRAC‐SNB)	1.34	18.1	NA	2% at <7	1.00	1.34	Likely underestimated
Lebanon (2003)^43^	470	15–45	Fasting	PBA (IA; Abbott AxSYM®)	1.36	19	NA	<1% at <7 [25% at <15]	1.00	1.36	Likely underestimated
Mexico (2012)^67^	4029	20–49	Fasting	PBA (IA; Abbott Architect)	1.14	26.3 (25.6–26.7)	NA	2% (1–3) at <9	1.29	0.89	Likely correct
Mongolia, regional (2001)^68^	205	17–51	Not reported	PBA (RIA; Bio‐Rad Quanta‐Phase II)	0.7	5.2	NA	88% (82–93) at <7	1.00	0.70	Likely overestimated
Mongolia (2004)^44^	408	15–49	Not reported	PBA (RIA; assay not specified	NA	Not reported	NA	13% at <3	0.43	NA	Cannot interpret (survey assay not known)
South Africa (2005)^64^	1676	16–35	Not reported	PBA (IA; Beckman Coulter® Access® and Bayer ADVIA Centaur®)	0.94 and 1.00	61.9 (58.9–64.8)	NA	<1% at <8 [2% at <13]	1.14	0.82 and 0.88	Cannot interpret (2 survey assays compare differently to MBA_C_)
Turkey, Edirne (not specified)^57^	704	12–17	Fasting	PBA (IA; DPC IMMULITE® 2000)	1.11	12.9	NA	16% at <7 [46% at 7–14]	1.00	1.11	Likely correct

a All surveys are nationally representative household‐based, cross‐sectional surveys except for surveys from Turkey (facility (school)‐based study representative at the first administrative level of Erdine),^57^ Iran (household and facility‐(school)‐based survey representative of the first administrative level of Golestan),^58^, ^59^ China, Shaanxi (first administrative level),^56^ Lebanon (nationwide convenience sample from health centers),^43^ and Mongolia, 2001 (regional, dzud affected and unaffected areas within country).^68^

b Ratio of survey assay results to MBA_C_ results. Calculated from proficiency testing data generated as close as possible to the time of the survey as the ratio between survey assay results and results obtained by the contemporary microbiologic assay calibrated with 5‐methyl‐THF and conducted at the Centers for Disease Control and Prevention.

c Values represent mean folate concentrations except as indicated: ^ǂ^geometric mean; ^#^median.

d Cutoff values represent those indicated in the corresponding survey report or publication; prevalence reported for a secondary cutoff is shown in square brackets; prevalence and cutoff values have been rounded to the nearest whole integer.

e Ratio of survey deficiency cutoff to MBA_C_ folate deficiency cutoff (7 nmol/L for serum folate and 305 nmol/L for RBC folate).

f Assay factor divided by cutoff factor.

g Prevalence estimates were considered likely correct if the calculated prevalence factor was >0.85 and <1.15, underestimated if the prevalence factor was ≥1.15, or overestimated if the prevalence factor was ≤0.85.

h The prevalence of folate deficiency in the Ecuador National Health and Nutrition Survey 2012 was not reported for women 12–49 years alone; however, it could be calculated for men and women 12–49 years (*n* = 14,009) combined as <1% at <9 nmol/L.

i Surveys in Fiji were conducted prior to and after initiation of mandatory fortification of wheat flour with folic acid at 1.6 ppm in 2004.^60^, ^61^

j Surveys in Iran were conducted prior to and after initiation of mandatory fortification of wheat flour.^58^, ^59^

5‐methyl‐THF, 5‐methyltetrahydrofolate; IA, immunoassay; MBA_C_, contemporary microbiological assay; PBA, protein binding assay; RBC, red blood cell; RIA, radioimmunoassay; NA, not applicable.

Disclaimer: The mention of specific companies or certain manufacturers’ products does not imply that they are endorsed or recommended by the WHO in preference to others of a similar nature that are not mentioned. Errors and omissions excepted, the names of proprietary products are distinguished by initial capital letters.

**Table 5:** Folate status of women of reproductive age based on RBC or serum/plasma folate samples collected in representative surveys^a^ in countries with high‐income economies

Country (year)	Sample size	Age range (years)	Fasting status	Survey assay	Assay factor^b^	Folate^c^ nmol/L (95% CI)	Prevalence of folate insufficiency (95% CI) and survey cutoff^d^ in nmol/L	Prevalence of folate deficiency (95% CI) and survey cutoff^d^ in nmol/L	Cutoff factor^e^	Prevalence factor^f^	Comments regarding the prevalence of folate deficiency^g^
**Assessment based on RBC folate concentration**											
Australia (2011–2012)^38^	2099	16–44	Casual	PBA (IA; Roche E170)	2.82	1647 1601#	<1% at <906	Not reported	NA	NA	Cannot interpret (deficiency prevalence not reported)
Canada, Newfoundland (2000–2001)^46^	204	19–44	Not reported	Not reported	NA	818ǂ (784–854)	Not reported	Not reported	NA	NA	Cannot interpret (deficiency prevalence not reported)
Canada (2007–2009)^72^	1162	15–45	Fasting	PBA (IA; Siemens IMMULITE® 2000)	1.62	1193# (1104–1282)	22% at <906	∼0% at <305	1.00	1.62	Likely underestimated
Ireland (2008–2010)^74^	368	18–50	Casual (79% fasting)	MBA_C_ (folic acid calibrator)	1.20	799#	64% at <906	Not reported	NA	NA	Cannot interpret (deficiency prevalence not reported)
New Zealand (2008/2009)^75^	Not reported	16–44	Casual	MBA_C_ (folic acid calibrator)	1.20	Not reported	73% at <906	4% at <340	1.11	1.08	Likely correct
Sweden (2010–2011)^45^	61	18–44	Casual	PBA (IA; Abbott Architect^TM^)	1.27	440#	100% at <906	Not reported	NA	NA	Cannot interpret (deficiency prevalence not reported)
United Kingdom (2000–2001)^34^	485	19–49	Casual	PBA (IA; Abbott IMx^TM^)	NA	652 581#	78% at <800 90% at <1000	5% at <350	1.15	NA	Cannot interpret (ratio of survey assay to MBA_C_ not known)
United Kingdom (2008/2009–2011/2012)^35^	600	16–49	Casual	MBA_C_ (5‐methyl‐THF calibrator)	1.00	614 535#	Not reported	11% at <340	1.11	0.90	Likely correct
United States (2007–2012)^77^	5670	12–49	Casual	MBA_C_ (5‐methyl‐THF calibrator)	1.00	992ǂ (973–1012)	23% (21–25) at <748	<1% at <305^h^ [6–13% at <624]	1.00	1.00	Likely correct
**Assessment based on serum/plasma folate concentration**											
Argentina (2004–2005)^69^	5322	10–49	Fasting	PBA (IA; Roche E170)	1.15	25.6 (24.5–27.7)	NA	<1% (0–2) at <7 [6% at 7–14]	1.00	1.15	Likely underestimated
Australia (2011–2012)^38^	2099	16–44	Casual	PBA (IA; Roche E170)	1.42	32.9 33.6#	NA	<1% at <7 [<1% at <11]	1.00	1.42	Likely underestimated
Austria (2010–2012)^70^	194	18–50	Not reported	PBA (RIA; MP Biomedicals SimulTRAC‐SNB)	1.34	19.4	NA	Not reported^i^	NA	NA	Cannot interpret (deficiency prevalence not reported)
Bahrain (2002)^71^	381	14–49	Not reported	Not reported	NA	24.7	NA	Not reported	NA	NA	Cannot interpret (deficiency prevalence not reported)
Canada, Newfoundland (2000–2001)^46^	204	19–44	Not reported	Not reported	NA	18.1ǂ (17.3–18.9)	NA	Not reported	NA	NA	Cannot interpret (deficiency prevalence not reported)
France (2006–2007)^73^	Not reported	18–49	Fasting	PBA (IA; assay not specified)	NA	Not reported	NA	7% (4–10) at <7	1.00	NA	Cannot interpret (survey assay not known)
Ireland (2008–2010)^74^	369	18–50	Casual (79% fasting)	MBA_C_ (folic acid calibrator)	1.20	25.2#	NA	Not reported	NA	NA	Cannot interpret (deficiency prevalence not reported)
New Zealand (2008/2009)^75^	976	15–50	Casual	MBA_C_ (folic acid calibrator)	1.20	28.0	NA	2% at <7	1.00	1.20	Likely underestimated
Spain, Madrid region (not specified)^76^	317	13–17	Fasting	PBA (IA; Roche E170)	1.42	7.8 (7.4–8.2)^j^	NA	24% at ≤5^j^	0.71	1.99	Likely underestimated
Sweden (2010–2011)^45^	66	18–44	Casual	PBA (IA; Abbott Architect^TM^)	1.14	14#	NA	Not reported	NA	NA	Cannot interpret (deficiency prevalence not reported)
United Kingdom (2000–2001)^34^	480	19–49	Casual	PBA (IA; Abbott IMx^TM^)	NA	21.4 20.1#	NA	6% at <10	1.43	NA	Cannot interpret (ratio of survey assay to MBA_C_ not known)
United Kingdom (2008/2009–2011/2012)^35^	616	16–49	Casual	HPLC–MS/MS	1.00	20 16.2#	NA	17% at <10	1.43	0.70	Likely overestimated
United States (1999–2010)^k^	9994	15–44	Casual	1999–2006: PBA (RIA; Bio‐Rad Quanta‐phase® II adjusted to MBA_C_ 2007–2010: MBA_C_ (5‐methyl‐THF calibrator)	1.00	NA	NA	<1% at <7 [2–6% at <14]	1.00	1.00	Likely correct

a All surveys are nationally representative, household‐based, cross‐sectional surveys, except for those indicated as follows: Canada 2000–2001 (evaluative survey conducted at the first administrative level of Newfoundland),^46^ and Spain (representative of the first administrative level of Madrid region/Comunidad de Madrid).^76^

b Ratio of survey assay results to MBA_C_ results. Calculated from proficiency testing data generated as close as possible to the time of the survey as the ratio between survey assay results and results obtained by the contemporary microbiologic assay calibrated with 5‐methyl‐THF and conducted at the Centers for Disease Control and Prevention.

c Values represent mean folate concentrations except as indicated: ^ǂ^geometric mean; ^#^median.

d Cutoff values represent those indicated in the corresponding survey report or publication; prevalence reported for a secondary cutoff is shown in square brackets; prevalence and cutoff values have been rounded to the nearest whole integer.

e Ratio of survey deficiency cutoff to MBA_C_ folate deficiency cutoff (7 nmol/L for serum folate and 305 nmol/L for RBC folate).

f Assay factor divided by cutoff factor.

g Prevalence estimates were considered likely correct if the calculated prevalence factor was >0.85 and <1.15, underestimated if the prevalence factor was ≥1.15, or overestimated if the prevalence factor was ≤0.85.

h The prevalence of folate deficiency from the United States 2007–2012 is based on weighted data from 1999 to 2010 NHANES surveys (*n* = 9968; ages 15–44) (personal communication, C. Pfeiffer).

i The prevalence of folate deficiency in the Austria 2010–2012 survey was not reported for women 18–50 years of age; however, it was reported for the larger age group of women 18–64 years of age as 2% <7 nmol/L and 19% 7–13 nmol/L.^70^

j Data for the Spain (Madrid) survey were only reported for boys (*n* = 145) and girls (*n* = 172) combined.^76^

k Personal communication, C. Pfeiffer.

5‐methyl‐THF, 5‐methyltetrahydrofolate; IA, immunoassay; MBA_C_, contemporary microbiological assay; PBA, protein binding assay; RBC, red blood cell; RIA, radioimmunoassay; NA, not applicable.

Disclaimer: The mention of specific companies or certain manufacturers’ products does not imply that they are endorsed or recommended by the WHO in preference to others of a similar nature that are not mentioned. Errors and omissions excepted, the names of proprietary products are distinguished by initial capital letters.

### Folate status

#### Summary of data from all economies

The present review identified 45 surveys conducted between 2000 and 2014 in 39 countries that assessed folate status in WRA and met our inclusion criteria. More than 70% of the surveys (*n* = 32) were conducted in countries with high‐income (*n* = 14) or upper‐middle‐income (*n* = 18) economies (Supplementary Fig. S1, online only). The remaining 13 surveys were conducted in countries with low‐income (*n* = 4) and lower‐middle‐income (*n* = 9) economies. In the four surveys from countries with low‐income economies, folate status was based solely on serum or plasma folate, and the reported prevalence of folate deficiency ranged from 7% to 79%. In countries with lower‐middle‐income economies, folate status was based solely on serum or plasma folate in six of the nine surveys, with the reported prevalence of deficiency ranging from <1% to 86%. Three surveys measured RBC folate, with the reported prevalence of deficiency ranging from 7% to 49% and the prevalence of folate insufficiency as a basis for NTD risk reported being 47% and 98% for Guatemala and Kyrgyzstan, respectively.^47^, ^48^

In countries with upper‐middle‐income economies, serum or plasma folate was assessed in 16 surveys, 15 of which reported a prevalence of folate deficiency ranging from <1% to 88%. Six surveys measured RBC folate, with the reported prevalence of deficiency ranging from <1% to 35%. Only Belize (49%) and Jordan (83%) reported the prevalence of folate insufficiency as a basis of NTD risk.^22^, ^41^, ^42^ In high‐income economies, serum or plasma folate status was assessed in 13 surveys, eight of which reported a prevalence of folate deficiency ranging from <1% to 24%. RBC folate status was measured in nine surveys, with five reporting a prevalence of folate deficiency ranging from ∼0% to 11% and seven reporting a prevalence of folate insufficiency ranging from <1% to 100%.

Additional details on folate status by income economy are presented below.

#### Countries with low‐income economies

The four surveys conducted in countries with low‐income economies (Table 2) were representative of the national population of adolescent girls in Afghanistan^37^ and WRA in Cambodia,^39^ Sierra Leone,^32^, ^33^ and Ethiopia.^49^ Folate status was based solely on serum or plasma folate concentration; therefore, the prevalence of folate insufficiency was not reported for any low‐income country. Three surveys used a PBA, and one survey did not report the type of assay used.

The 2013 Afghanistan National Nutrition Survey reported a 7% prevalence of folate deficiency at a cutoff of <7 nmol/L.^37^ Because the folate assay used in the Afghanistan survey was not reported, we do not know whether this prevalence correctly represents deficiency. The 2014 Cambodian Micronutrient Survey reported an 18% prevalence of folate deficiency at <10 nmol/L in WRA.^39^ The ratio of the survey assay's results to the MBA_c_ results from proficiency testing (assay factor) was 1.59, while the ratio of the survey cutoff to the deficiency cutoff (cutoff factor) was 1.43 (10/7), resulting in a prevalence factor of 1.11 (1.59/1.43) (Table 2). Because the prevalence factor was >0.85 but <1.15, it was concluded that the prevalence was likely a correct estimate of deficiency. The 2013 Sierra Leone Micronutrient Survey reported a prevalence of folate deficiency in WRA of ∼80% at <10 nmol/L^32^, ^33^ and used the same assay and cutoff as the Cambodian survey; thus, the prevalence was also likely a correct estimate of deficiency. The 2005 Ethiopian national assessment (9 of 11 regions) of folate status in WRA reported that nearly 50% of WRA had serum folate values ≤9 nmol/L.^49^ The calculated prevalence factor was 0.89, thus we concluded that Ethiopia reported the prevalence of nearly 50% serum folate deficiency which was likely a correct estimate (Table 2).

#### Countries with lower‐middle‐income economies

Of the nine surveys conducted in countries with lower‐middle‐income economies (Table 3), two reported both RBC and serum folate concentrations (Guatemala^47^, ^50^ and the Philippines^51^) and one reported RBC concentration alone (from dried blood spots; Kyrgyzstan^48^). In six other surveys, folate status was based solely on serum or plasma folate (Bangladesh,^52^ Cameroon,^53^ Côte d'Ivoire,^31^ Georgia,^54^ Uzbekistan,^36^ and Vietnam^55^).

In Guatemala^47^, ^50^ and Kyrgyzstan,^48^ prevalence estimates for folate insufficiency and deficiency are likely correct estimates because they used an MBA_C_ method and assay‐matched cutoffs (Table 3 and Supplementary Table S1, online only). In the 2009–2010 Guatemala National Micronutrients Survey (ENMICRON), the prevalence of folate insufficiency among WRA was 47%, while the prevalence of folate deficiency was 7% and <1% for RBC folate and serum folate, respectively.^47^, ^50^ In Kyrgyzstan, almost all WRA (98%) were classified as folate insufficient, and about half (49%) were classified as folate deficient based on RBC folate.^48^

In the 2008 Philippines 7th National Nutrition Survey, RBC and serum folate concentrations were determined using a PBA and the prevalence of folate deficiency was reported as 21% using a cutoff of <397 nmol/L for RBC folate, and was reported as 39% using a cutoff of <7 nmol/L for serum folate (Table 3).^51^ Insufficiency was not reported. The reported prevalence of serum folate deficiency likely underestimated the true prevalence of deficiency. However, because no proficiency testing data are available for RBC folate, it is difficult to assess possible bias in the reported prevalence based on RBC folate.

Serum or plasma folate was assessed in six additional surveys within countries with lower‐middle‐income economies with four using an MBA_C_ method (Côte d'Ivoire,^31^ Georgia,^54^ Uzbekistan,^36^ and Vietnam^55^). In two of these studies Côte d'Ivoire^31^ and Uzbekistan,^36^ the prevalence of deficiency was likely overestimated. In the 2007 Côte d'Ivoire survey, over 80% of WRA had serum folate <10 nmol/L.^31^ Blood samples were stored at –25 °C for approximately 9 months before the analysis, which may have caused some folate losses (Supplementary Table S1, online only); therefore, the prevalence of deficiency according to any cutoff may be overestimated. The 2008 national survey in Uzbekistan (Large Country‐Lot Quality Assurance Sampling survey) reported a 29% folate deficiency in WRA (serum folate <10 nmol/L).^36^ The 2009 Georgia National Nutrition Survey^54^ and the 2010 Vietnam Micronutrient Study^55^ reported that, respectively, 25% and 3% of WRA were folate deficient (serum or plasma folate <7 nmol/L). The calculated prevalence factor for both studies was 1.20, suggesting that the prevalence in each study was likely underestimated (Table 3).

National surveys conducted in Bangladesh (Micronutrients Status Survey 2011–2012)^52^ and Cameroon (2009)^53^ assessed serum folate using a PBA. In Bangladesh, the estimate of 9% folate deficiency (serum folate <7 nmol/L) in WRA^52^ was likely an underestimation since the PBA method used in this survey is reported to produce higher results than the CDC MBA_C_ method (Table 3 and Supplementary Table S1, online only). In Cameroon, the prevalence of folate deficiency was estimated to be 17% in WRA based on plasma folate <10 nmol/L.^53^ The calculated prevalence factor suggests that the overall reported prevalence for folate deficiency was likely correct (Table 3).

#### Countries with upper‐middle‐income economies

Of the 18 surveys in 16 countries with upper‐middle‐income economies (Table 4), 13 surveys were representative of the national population of WRA (the Lebanon survey was nationally representative of only women attending government health centers across the country),^43^ one survey was representative of the regional level (Mongolia),^44^ and four were representative of the first administrative level (Shaanxi province, China;^56^ Edirne province, Turkey;^57^ and two from Golestan Province, the Islamic Republic of Iran^58^, ^59^). Four of the surveys reported both RBC and serum folate in WRA, two reported only RBC folate, and the remaining 12 reported serum or plasma folate alone. For two countries (Fiji^60^, ^61^ and the Islamic Republic of Iran^58^, ^59^), surveys were conducted both pre‐ and postfortification, reporting only serum folate data.

Of the six countries reporting RBC folate, four used an MBA method (Belize,^22^ Dominican Republic,^62^ Iraq,^63^ and Jordan^41^, ^42^), and two used a PBA method (Ecuador^40^ and South Africa^64^). Only two surveys (Belize^22^ and Jordan^41^, ^42^) reported prevalence estimates for folate insufficiency and both countries used appropriate assay‐matched cutoffs (Table 4); therefore, these prevalence estimates are likely correct. Approximately 50% and 83% of WRA were classified as folate insufficient based on results of the Belize 2011 National Micronutrient Survey^22^ and the Jordan 2010 National Micronutrient Survey,^41^, ^42^ respectively. All six of these surveys reported prevalence estimates for folate deficiency based on RBC folate, and four presented estimates for folate deficiency based on serum folate. The Dominican Republic 2009 National Micronutrients Survey^62^ reported a relatively low prevalence of folate deficiency (6% for RBC folate <317 nmol/L; 2% for serum folate <7 nmol/L) as did the survey in Jordan (10% for RBC folate <317 nmol/L)^41^, ^42^ although these were all likely underestimations. The Belize 2011 survey^22^ reported a 35% prevalence of folate deficiency based on RBC folate <624 nmol/L and an 11% prevalence based on serum folate <14 nmol/L, both likely overestimations. In the Iraq National Micronutrient Survey 2011–2012, a 19% prevalence of folate deficiency was reported for WRA based on RBC folate <342 nmol/L.^63^ The prevalence factor was 0.89 in this survey, indicating that the reported prevalence estimate is likely correct.

The remaining two surveys (Ecuador^40^ and South Africa^64^) that measured RBC folate concentrations in an upper‐middle‐income economy used a PBA and both also measured serum folate. In Ecuador's 2012 National Health and Nutrition Survey, a low prevalence of folate deficiency in WRA (<1% for RBC folate <342 nmol/L) was reported,^40^ although this is likely an underestimation based on the prevalence factor (1.44) (Table 4). The prevalence of deficiency based on serum folate was not reported; however, the mean fasting concentration was 37 nmol/L, which is similar to that in the U.S. postfortification.^19^ Since the assay factor was 1.03, it is likely that this represents the correct mean serum folate for Ecuador (Table 4). In the South Africa 2005 National Food Consumption Survey‐Fortification Baseline, the reported prevalence of folate deficiency was low based on both RBC (2% at <616 nmol/L) and serum folate (1% at <8 nmol/L).^64^ Because folate was assayed by two different methods and the source of the cutoffs used was not reported, we could not comment on any potential bias in this survey.

As mentioned previously, serum or plasma folate alone was assessed in 10 surveys within countries with upper‐middle‐income economies. One survey assessed serum folate by MBA_C_ (the 2013 Azerbaijan Nutrition Survey) and 35% of WRA were classified as folate deficient (plasma folate <10 nmol/L)^65^ although this was likely an overestimation (Table 4). Several types of PBA methods were used for the remaining surveys (Shaanxi, China,^56^ Taiwan, China,^66^ Fiji,^60^, ^61^ the Islamic Republic of Iran,^58^, ^59^ Lebanon,^43^ Mexico,^67^ Mongolia,^44^, ^68^ and Edirne, Turkey^57^). Deficiency prevalences at a cutoff of <7 nmol/L reported for Shaanxi, China (15%)^56^ and Lebanon (<1%)^43^ are likely underestimated with prevalence factors ≥1.15 (Table 4 and Supplementary Table S1, online only). In contrast, the reported low prevalence of folate deficiency in the National Health and Nutrition Survey in Taiwan 2005–2008 (2% <7 nmol/L)^66^ and the 2012 Mexican National Health and Nutrition Survey (ENSANUT) (2% <9 nmol/L)^67^ and the reported 16% prevalence of folate deficiency (<7 nmol/L) in the subnational survey conducted in Edirne, Turkey^59^ are likely correct based on prevalence factors for the surveys of >0.85 and <1.15.

In the 2001 Mongolian regional survey assessing the nutrition consequences of the *dzud* (severe winter weather), serum folate was measured using the Bio‐Rad radioimmunoassay (RIA) in WRA from areas severely affected by the *dzud* and areas that were only slightly affected or unaffected by the *dzud*. There were no differences in mean serum folate of women in the two areas, but overall, 88% of women were reported to be folate deficient (serum folate <7 nmol/L),^68^ which is likely an overestimation (Table 4). The 2004 3rd National Nutrition Survey of Mongolia reported a prevalence of folate deficiency in WRA of 13% (serum folate <3 nmol/L).^44^ A RIA was used to measure serum folate, but the results could not be interpreted because the manufacturer of the RIA was not reported.

Serum folate was assessed in pre‐ and postfortification surveys in two countries using PBA methods. In Fiji, national surveys were conducted before (2004) and after (2010) initiation of mandatory fortification of wheat flour with iron, zinc, and B vitamins (folic acid, thiamine, riboflavin, and niacin) in 2005.^60^, ^61^ Mean serum folate concentrations were 18 nmol/L prefortification and 26.6 nmol/L postfortification (Table 4).^60^, ^61^ A marked improvement in folate deficiency prevalence was also observed (1% in 2010 as compared to 8% in 2004 (serum folate <10 nmol/L)). Given that the prevalence factors for the surveys were 0.99 and 0.84 in 2010 and 2004, respectively, the reported prevalence of deficiency is likely correct for 2010 but likely overestimated for 2004 (Table 4). In the Islamic Republic of Iran, mandatory wheat flour fortification with folic acid (1.5 ppm) started in 2006, just after the prefortification survey.^58^ A postfortification survey took place in 2008,^59^ 18 months after implementation. Both surveys took place in the Golestan Province. The prevalence of folate deficiency (serum folate <7 nmol/L) was 14% prefortification and 2% postfortification but is likely underestimated in both surveys (Table 4).

#### Countries with high‐income economies

There were 14 surveys conducted within 12 countries with high‐income economies (Argentina,^69^ Australia,^38^ Austria,^70^ Bahrain,^71^ Canada,^46^, ^72^ France,^73^ Ireland,^74^ New Zealand,^75^ Spain,^76^ Sweden,^45^ the United Kingdom,^34^, ^35^ and the United States^77^). Two surveys each were conducted in Canada^46^, ^72^ and the United Kingdom.^34^, ^35^ Additionally, the U.S. National Health and Nutrition Examination Survey (NHANES) involves a series of continuous 2‐year survey cycles.^77^ Surveys were representative of the national populations of WRA, except for the two surveys that were conducted at the first administrative level (WRA of Newfoundland and Labrador province in Canada (2000–2001)^46^ and in adolescent boys and girls of the Madrid Region (Comunidad de Madrid), Spain).^76^ Six surveys reported both RBC and serum or plasma folate concentrations in WRA, two reported only RBC folate concentrations, and six reported only serum or plasma folate concentrations (Table 5).

Of the nine surveys reporting RBC folate concentrations, only four (Ireland 2008–2010,^74^ New Zealand 2008–2009,^75^ the United Kingdom 2008–2012,^35^ and the United States 2007–2012^77^) used an MBA method. Three of these four surveys reported the prevalence of folate insufficiency (RBC folate concentrations < 906 nmol/L, or the assay‐matched equivalent) in WRA: 23% in the United States,^77^ 64% in Ireland,^74^ and 73% in New Zealand.^75^ In these three countries, the prevalence of deficiency based on RBC folate was reported in only the United States (<1%) and New Zealand (4%); both values were considered being likely correct (Table 5). The prevalence of folate insufficiency was not reported in the UK 2008–2012 National Diet and Nutrition Survey Rolling Programme (NDNS RP) Years 1–4 (September 2008–December 2011); however, 11% of WRA were folate deficient (RBC folate <340 nmol/L) as assessed using MBA_C_ 35 and this value was considered being likely correct (Table 5).

Of the remaining five surveys reporting RBC folate concentrations, four (Australia,^38^ Canada 2007–2009,^72^ Sweden,^45^ and the United Kingdom 2000–2001^34^) used a PBA method and reported the prevalence of folate insufficiency in WRA. The prevalence of folate insufficiency (RBC folate <906 nmol/L) in WRA was <1% in the Australian Health Survey 2011–2012,^38^ 22% in the Canadian Health Measures Survey 2007–2009,^72^ and 100% in Sweden's Riksmaten Adults 2010–2011 survey.^45^ Because of the high calculated prevalence factors in the Australian (2.33) and Canadian (1.34) surveys, the prevalence of folate insufficiency is likely underestimated in these surveys (data not shown). On the other hand, the prevalence factor in the Swedish survey was 1.05 and therefore the 100% insufficiency prevalence estimate derived using a cutoff of <906 nmol/L may be appropriate. In the 2000–2001 UK National Diet and Nutrition Survey (NDNS) of adults aged 16–64 years, RBC folate was measured using a PBA.^34^ This survey reported that among women aged 19–49 years, 78% had RBC folate concentrations <800 nmol/L and 90% had RBC folate concentrations <1000 nmol/L. Lack of information on how this method compares to the CDC MBA_C_ prevents us from interpreting these prevalence estimates. The last survey that measured RBC folate was conducted in Newfoundland, Canada in 2000–2001.^46^ This survey did not report the assay used, neither did it report a prevalence of folate deficiency/insufficiency; however, the (geometric) mean RBC and serum folate concentration of WRA were 818 and 18.1 nmol/L, respectively.

Serum or plasma folate was assessed in 13 surveys within 12 countries with high‐income economies. The surveys used various assays, including MBA, HPLC–MS/MS, and different PBAs. Two surveys did not report the assay used to assess serum folate (Bahrain^71^ and Canada (Newfoundland)^46^) and two did not report the specific assay method (France^73^ and the UK 2000–2001 NDNS^34^). The Argentina 2004–2005 National Nutrition and Health Survey (ENNyS)^69^ and the Australian Health Survey 2011–2012^38^ reported a <1% prevalence of folate deficiency (<7 nmol/L) using a PBA. Both surveys had a prevalence factor ≥1.15, indicating that the reported prevalence of folate deficiency was likely underestimated (Table 5 and Supplementary Table S1, online only). The 2008–2009 New Zealand Adult Nutrition Survey reported a 2% prevalence of folate deficiency (serum folate <7 nmol/L) using MBA_C_.^75^ Given a prevalence factor of 1.20, the reported prevalence is likely underestimated (Table 5). The Spanish survey was conducted in a school‐based population of adolescents 13–17 years in the Madrid Region.^76^ Serum folate was assessed using a PBA and a prevalence of 24% at ≤5 nmol/L was reported for girls and boys combined (172 girls and 145 boys; no data presented for girls only). The prevalence factor for this survey was 1.99, indicating that the reported prevalence of folate deficiency was likely underestimated (Table 5).

The U.S. NHANES 1999–2010 surveys used the Bio‐Rad RIA from 1999 to 2006 and the MBA_C_ from 2007 to 2010.^77^ The data assessed using the RIA were adjusted to the MBA_C_ and then combined with data from 2007 to 2010, which resulted in a <1% prevalence of serum folate deficiency (personal communication, C. Pfeiffer). Because the prevalence factor was 1.00 in this survey, the reported prevalence is expected to correctly represent folate deficiency (Table 5). The 2006 French Nutrition and Health Survey reported a 7% prevalence of folate deficiency (serum folate <7 nmol/L);^73^ however, without knowing which specific method was used, the prevalence cannot be interpreted. The UK 2000–2001 NDNS of adults aged 16–64 years and the UK NDNS RP Years 1–4 2008–2012 surveys reported the prevalence of folate deficiency (serum folate <10 nmol/L) as 6% using a PBA^34^ and 17% using HPLC–MS/MS,^35^ respectively. The PBA used in the 2000–2001 NDNS was not specified and therefore the prevalence cannot be interpreted. The prevalence factor for the NDNS RP years 1–4 2008–2012 survey was 0.70, indicating that the reported prevalence of folate deficiency of 17% is likely an overestimate (Table 5). The remaining five surveys (Austrian Nutrition Report 2010–2012,^70^ Bahrain 2002,^71^ Newfoundland, Canada,^46^ Irish National Adult Nutrition Survey 2008–2010,^74^ and Sweden's Riksmaten Adults 2010–2011 survey^45^) did not report a prevalence of folate deficiency based on serum folate.

## Discussion and conclusions

It has been over a decade since the folate status of WRA worldwide has been reviewed. Although the number of nationally representative surveys (*n* = 38) in WRA was greater in this review relative to the previous report (*n* = 5),^15^ a striking paucity of data remains. The majority of surveys on which these estimates of folate status are based were conducted in countries with high‐income economies, emphasizing the scarcity of folate status data in the world's most vulnerable WRA. This is particularly the case in relation to estimating folate insufficiency since RBC folate was only measured in 18 representative surveys, of which only three were conducted in countries with low‐ or lower‐middle‐income economies. Even given the limited data available, however, it is clear that folate deficiency and insufficiency are problems in many countries around the world. The prevalence of folate deficiency was >20% in many countries with lower income economies but was typically <5% in countries with higher income economies. Only 11 of the 18 surveys measuring RBC folate reported the prevalence of folate insufficiency, which was >40% in most countries.

Nonetheless, even with the increase in the number of available surveys assessing folate status in WRA, the diversity of assays used and the use of nonassay matched cutoffs to define folate deficiency and insufficiency make the interpretation of results difficult and complicate the direct comparison of folate status of WRA within and between countries. Our review focused on these challenges and illustrates how differences in folate assay methodology and selection of cutoffs impact conclusions related to the prevalence of folate deficiency and insufficiency. Rather than grouping assays used in each survey into a few major types of assays (i.e., MBA, RIA, IA, and HPLC–MS/MS), we separately assessed each assay's performance in relation to a comparison assay based on proficiency testing data generated for the assays around the time of the survey. We also assessed the cutoffs used in the survey in relation to the assay‐matched recommended cutoffs for defining folate deficiency and insufficiency. This detailed approach is a major strength of this paper as it results in an improved accuracy in the interpretation of the data. Had we generalized the data to a few major types of assays, we would have introduced significant bias because different assays under the same assay type can generate very different results relative to a comparison assay.

Despite our attempt to make these data more comparable, our approach using proficiency testing data still has many limitations, including (1) the average percent difference between assays does not capture the sometimes large among‐sample variability or a concentration‐dependent bias,^21^, ^78^ (2) assay comparisons used smaller than desirable numbers of proficiency testing samples in some cases; (3) data on assay comparisons sometimes came from several years before the survey; and (4) proficiency testing samples may behave differently than native patient samples because they may have to be modified to improve their stability or allow generation of large volumes of material.^79^ While in the latter case the direction of the bias is not predictable, this weakness is unlikely to have affected our interpretation because the majority of assay comparisons were based on proficiency data from the UK NEQAS Haematinics program, which uses largely unmodified materials.

Although 35 surveys reported serum or plasma folate, only 29% could be interpreted as likely correct, whereas of the 11 surveys reporting RBC folate, the majority (73%) were interpreted as likely correct. Our findings indicate the importance of using appropriate assays and cutoffs to describe blood folate status; the bulk of data collected to date cannot be interpreted correctly, as they are currently reported. While this report did not focus on folate deficiency based on rising homocysteine concentrations, the report of the Belize 2011 National Micronutrient Survey^22^ assessed that type of folate deficiency using RBC folate provides an excellent illustration of how different conclusions can be drawn if the assay‐mismatched cutoffs are used. The use of the assay‐mismatched cutoff (<340 nmol/L) resulted in a prevalence of 7% deficiency, while the use of the assay‐matched cutoff (<624 nmol/L) resulted in a prevalence of 35%, a fivefold increase. Furthermore, data from a few surveys^31^, ^33^, ^34^, ^35^, ^36^ identified the problem of likely over‐ or underestimating folate deficiency because of potential folate losses due to suboptimal long‐term sample storage. These data provide strong evidence supporting the importance of the proper collection and storage of blood samples, timely assessment, and the use of assay‐matched cutoffs to define folate deficiency and insufficiency.

Folic acid intervention programs, such as fortification of staple foods and targeted supplementation, may have been implemented in some of the populations surveyed; however, coverage of these interventions was not always known, and discussion of these programs and their potential impact on blood folate status was beyond the scope of the current review. It has been established that well‐implemented folic acid intervention programs (i.e., those with demonstrated industry compliance, following standards aligned with WHO guidelines) are effective in increasing blood folate status and decreasing NTD prevalence.^5^, ^80^, ^81^, ^82^ A future analysis examining the characteristics of intervention programs in those countries with blood folate data could highlight implementation or coverage gaps that could be addressed to ensure the adequate improvement of folate status and thus maximum NTD prevention.

Moreover, it is important to note that examining only the overall prevalence of folate deficiency or insufficiency, as reported in our review, may ignore population subgroups that remain at increased risk of having an NTD‐affected pregnancy due to folate insufficiency. As such, even in those surveys reporting a lower prevalence of folate deficiency or insufficiency, it is plausible that vulnerable population subgroups may be at increased risk. For instance, the national prevalence of RBC folate insufficiency in Guatemala was reported as 47%, but when stratified by region, folate insufficiency ranged from a low of 19% in the Metropolitan region to a high of 81% in the Northern region.^47^ Additionally, the prevalence of folate insufficiency varied greatly by wealth index with a reported prevalence of 27% in those with a high wealth index and 68% in those with a low wealth index. Hence, when considering folate status, it is important to stratify data by key variables such as a region, urban/rural areas, ethnicity, and income to better understand if there are specific population subgroups who may be at higher risk than others.

In summary, the prevalence of folate deficiency was >20% in many countries with lower income economies but was typically <5% in countries with higher income economies. RBC folate was measured in 18 surveys but only 11 reported the prevalence of folate insufficiency, which was >40% in most countries. Despite the data limitations described, folate deficiency and insufficiency are of major concern in WRA in many countries globally, and these data warrant consideration of the appropriate folic acid intervention approaches to prevent anemia and NTDs. Our review provides additional evidence illustrating why folate status should be measured using an MBA as recommended by the WHO,^1^, ^24^ that is harmonized with regard to common reagents and protocols along with assay‐matched cutoffs to define folate deficiency and insufficiency. When reporting survey results, it is essential to fully describe the methods of blood sampling, storage, and transport; type of assay used; use of internal and external quality controls; and participation in external quality assurance programs. Furthermore, in addition to reporting means and variance stratified by key population characteristics, it is suggested to present values using cutoffs established for estimating the prevalence of megaloblastic anemia and risk of NTDs.

## Disclaimer

This paper was developed in support of the Technical Consultation: Folate Status in Women and Neural Tube Defect Prevention, convened by the Micronutrient Forum and supported through Nutrition International by a grant provided by the Bill & Melinda Gates Foundation. Additional manuscripts related to this Technical Consultation have been published as the special issue “Folate Status in Women and Neural Tube Defect Risk Reduction” in Ann. N.Y. Acad. Sci. 1414: 1–136 (2018), The authors alone are responsible for the views expressed in this paper; they do not necessarily represent the views, decisions, or policies of the institutions with which they are affiliated or the decisions, policies, or views of the Micronutrient Forum, the sponsors, publisher, or editorial staff of *Annals of the New York Academy of Sciences*.

## Author contribution

The authors acknowledge Joanna Taliano for conducting the systematic search of the literature and Taylor Snyder for assisting with data extraction. L.M.D.R., B.L.T., and L.M.R. were responsible for the initial conception of this review and L.B.B. and L.M.R. were responsible for expanding the scope. L.M.D.R., B.L.T., L.M.R., and A.M.C. were responsible for developing the search strategy. A.M.C., J.R., H.R., and E.C.W. were responsible for selecting the surveys to include, with input from L.M.R. L.M.R., D.B.H., L.B.B., and C.M.P. were responsible for data extraction. C.M.P. was responsible for the critical assessment of blood collection method and reporting of blood folate data. L.B.B., C.M.P., D.B.H., and L.M.R. were responsible for summarizing and interpreting the results and accept responsibility for the integrity of the data. L.B.B., L.M.R., D.B.H., C.M.P., A.M.C., and A.P.W. drafted the final review. All authors read and approved the final version of the manuscript.

## Competing interests

The authors declare no competing interests.

## Supporting information


**Appendix S1**. Full search strategy.

**Appendix S2**. Assessment of possible bias in folate concentrations and prevalence estimates due to the choice of assay and cutoff(s).

**Table S1**. Data on assay performance and specimen handling and storage to inform the interpretation of results.

**Figure S1**. Surveys included in this article grouped by income category and indicating sample sizes available for serum and RBC folate. Gray bars indicate serum/plasma folate and black bars indicate RBC folate.

**Figure S2**. Reported mean serum/plasma folate concentrations in women of reproductive age by the survey, indicating an interpretation of the data based on the assay used in the survey relative to the CDC MBA_C_.

**Figure S3**. Reported mean red blood cell folate concentrations in women of reproductive age by the survey, indicating an interpretation of the data based on the assay used in the survey relative to the CDC MBA_C_.
