# Supplementary material for: Global folate status in women of reproductive age: a systematic review with emphasis on methodological issues
Source: Ann N Y Acad Sci. 2018 Sep 21;1431(1):35–57. doi: 10.1111/nyas.13963 (PMC6282622; doi:10.1111/nyas.13963)
Supplement: Supplementary file 1 — Appendix S1. Full search strategy. [file NYAS-1431-35-s005.docx]

**Supporting information**

**Appendix S1. Full search strategy**

1. *MEDLINE(Ovid) 1946- (searched up to and including 30 June 2017)*

| **Search statement** | **Search terms** |
| --- | --- |
| 1 | (folate* or folic) ADJ5 (blood or deficien* or plasma or "red blood cell*" or RBC or serum or status* or concentration) |
| 2 | exp Folic Acid/bl or Folic Acid Deficiency/ or vitamin b 12 deficiency/ or anemia, pernicious/ or "pernicious anemia" or ("vitamin B12" ADJ3 deficien*) |
| 3 | 1 or 2 |
| 4 | Prevalence/ or prevalen*.ti. |
| 5 | geography/ or geography, medical/ or "global health" or World Health/ or "world health".ti. or World Health Organization/ |
| 6 | (national and survey*) or (population and prevalence) |
| 7 | exp geographic locations/ and epidemiology.fs. |
| 8 | exp population groups/ or population.ti. |
| 9 | epidemiological monitoring/ or health status indicators/ or health surveys/ or mass screening/ or nutrition assessment/ or nutrition surveys/ or exp population surveillance/ or program evaluation/ or ((program or programs) ADJ5 evaluat*) |
| 10 | 4 or 5 or 6 or 7 or 8 or 9 |
| 11 | 3 and 10 |
| 12 | Exp animals/ not exp humans/ |
| 13 | 11 not 12 |
| 14 | Remove duplicates |

1. *PubMed* *(searched up to and including 30 June 2017)*

| **Search statement** | **Search terms** |
| --- | --- |
| 1 | (folate*[Title/Abstract] OR folic[Title/Abstract]) AND (blood[Title/Abstract] OR deficien*[Title/Abstract] OR plasma[Title/Abstract] OR "red blood cell*"[Title/Abstract] OR RBC[Title/Abstract] OR serum[Title/Abstract] OR status*[Title/Abstract] OR concentration[tiab]) |
| 2 | "pernicious anemia"[Title/Abstract] OR ("vitamin B12"[Title/Abstract] AND deficien*[Title/Abstract]) |
| 3 | #1 OR #2 |
| 4 | prevalen*[Title/Abstract] |
| 5 | "global health"[Title/Abstract] OR "world health"[Title/Abstract] |
| 6 | national[Title/Abstract] AND survey*[Title/Abstract] |
| 7 | (geograph*[Title/Abstract] OR location*[Title/Abstract] OR country[Title/Abstract] OR countries[Title/Abstract] OR region*[Title/Abstract] OR city[Title/Abstract] OR cities[Title/Abstract]) AND epidemiol*[Title/Abstract] |
| 8 | population[Title/Abstract] |
| 9 | "epidemiological monitoring"[Title/Abstract] OR "health status"[Title/Abstract] OR "health survey*"[Title/Abstract] OR "mass screening"[Title/Abstract] OR "nutrition assessment"[Title/Abstract] OR "nutrition survey"[Title/Abstract] OR "program evaluation"[Title/Abstract] |
| 10 | #4 OR #5 OR #6 OR #7 OR #8 OR #9 |
| 11 | #3 AND #10 |
| 12 | #11 NOT MEDLINE[sb] |
| 13 | Limit 2000-2017 |

1. *Embase (Ovid) 1996-* *(searched up to and including 30 June 2017)*

| **Search statement** | **Search terms** |
| --- | --- |
| 1 | (folate* or folic) ADJ5 (blood or deficien* or plasma or "red blood cell*" or RBC or serum or status* OR concentration ) |
| 2 | folic acid blood level/ or folic acid deficiency/ or cyanocobalamin deficiency/ or pernicious anemia/ or "pernicious anemia".mp. or ("vitamin B12" adj3 deficien*) |
| 3 | 1 or 2 |
| 4 | prevalence/ or seroprevalence/ or prevalen*.ti. |
| 5 | geography/ or exp medical geography/ or "global health" or "world health".ti. or World Health Organization/ |
| 6 | (national and survey*) or (population and prevalence) |
| 7 | exp geographic names/ and ep.fs. |
| 8 | exp population groups/ or population.ti. |
| 9 | disease surveillance/ or epidemiological monitoring/ or health status indicator/ or health survey/ or mass screening/ or nutritional assessment/ or nutritional status/ or "nutrition survey*" or exp program evaluation/ or ((program or programs) ADJ5 evaluat*) |
| 10 | 4 or 5 or 6 or 7 or 8 or 9 |
| 11 | 3 and 10 |
| 12 | Exp animal/ not exp human/ |
| 13 | 11 not 12 |
| 14 | Limit 13 to 2000-current; exclude medline journals |

1. *Scopus*

| **Search statement** | **Search terms** |
| --- | --- |
| 1 | INDEXTERMS((folate* W/5 blood) or (folate* W/5 deficien*) or (folate* W/5 plasma) or (folate* W/5 "red blood cell*") or (folate* W/5 RBC) or (folate* W/5 serum) or (folate* W/5 status*) or (folate* W/5 concentration) or (folic W/5 blood) or (folic W/5 deficien*) or (folic W/5 plasma) or (folic W/5 "red blood cell*") or (folic W/5 RBC) or (folic W/5 serum) or (folic W/5 status*) or (folic W/5 concentration)) |
| 2 | INDEXTERMS("pernicious anemia" or ("vitamin B12" W/3 deficien*)) |
| 3 | #1 or #2 |
| 4 | TITLE(prevalen* OR "world health" OR global OR population) |
| 5 | INDEXTERMS("global health" or "world health organization" or city or cities or country or countries or geograph* or local* or province* or region* or town or towns) |
| 6 | INDEXTERMS((national and survey*) or (population and prevalence)) |
| 7 | INDEXTERMS((epidemiolog* W/5 monitor*) or "health status" or "health survey*" or "mass screening" or (nutrition* W/3 assess*) OR (nutrition* W/3 survey*) or "population surveillance" or (program* W/5 evaluat*)) |
| 8 | #4 or #5 or #6 or #7 |
| 9 | (#3 and #8) AND NOT INDEX(medline) |
| 10 | Limit 2000-2017 |

1. *CINAHL*

| **Search statement** | **Search terms** |
| --- | --- |
| 1 | (folate* OR folic) N5 (blood OR deficien* OR plasma OR "red blood cell*" OR RBC OR serum OR status* OR concentration) |
| 2 | (MH "Folic Acid+/BL") OR (MH "Folic Acid Deficiency") OR (MH "Vitamin B12 Deficiency+") OR "pernicious anemia" OR ("vitamin B12" N3 deficien*) |
| 3 | S1 OR S2 |
| 4 | (MH "Prevalence") OR (TI prevalen*) |
| 5 | "global health" OR (TI "world health") OR (MH "World Health") OR (MH "World Health Organization") |
| 6 | (national AND survey*) OR (population AND prevalence) |
| 7 | (MH "Geographic Locations+") |
| 8 | (MH "Population") OR (MH "Population Surveillance") OR (MH "Population Characteristics+") OR (TI population) |
| 9 | (MH "Epidemiological Research") OR (MH "Seroprevalence Studies") OR (MH "Health Status Indicators") OR (MH "Health Status") OR (MH "Health Status Disparities") OR (MH "Surveys") OR (MH "Health Screening") OR "mass screening" OR (MH "Nutritional Assessment") OR (MH "Program Evaluation") OR ((program or programs) N5 evaluat*) |
| 10 | S4 or S5 or S6 or S7 or S8 or S9 |
| 11 | S3 and S10  **Limiters** - Exclude MEDLINE records |

1. *Global Health (Ovid) 1973-*

| **Search statement** | **Search terms** |
| --- | --- |
| 1 | (folate* or folic) ADJ5 (blood or deficien* or plasma or "red blood cell*" or RBC or serum or status* or concentration) |
| 2 | (Folic Acid/ and (blood or deficien* or plasma or "red blood cell*" or RBC or serum or status* )) or Folic Acid Deficiency/ |
| 3 | 1 or 2 |
| 4 | disease prevalence/ or disease distribution/ or seroprevalence/ or prevalen*.ti. |
| 5 | geography/ or exp countries/ or exp regions/ or "global health" or "world health".ti. or WHO/ |
| 6 | (national and survey*) or (population and prevalence) |
| 7 | exp ethnic groups/ or serological surveys/ or population.ti. |
| 8 | epidemiological surveys/ or "health status" or "health survey*" or screening/ or nutritional assessment/ or exp nutrition surveys/ or surveillance/ or program evaluation/ or ((program or programs) ADJ5 evaluat*) |
| 9 | 4 or 5 or 6 or 7 or 8 |
| 10 | 3 and 9 |
| 11 | Limit 2000-current |

1. *POPLINE*

| **Search statement** | **Search terms** |
| --- | --- |
| 1 | "Folic Acid" OR "folate concentration" OR "blood folate" OR "folate deficiency" OR "folate status" OR "serum folate" OR "vitamin B12 deficiency" OR "red blood cell folate" |

|  |  |
| --- | --- |

1. *Cochrane Central Register of Controlled Trials*

| **Search statement** | **Search terms** |
| --- | --- |
| 1 | (folate* or folic) near/5 (blood or deficien* or plasma or "red blood cell*" or RBC or serum or status* OR concentration):ti,ab,kw |
| 2 | MeSH descriptor: [Folic Acid] explode all trees and with qualifier(s): [Blood - BL] |
| 3 | MeSH descriptor: [Folic Acid Deficiency] explode all trees |
| 4 | MeSH descriptor: [Vitamin B 12 Deficiency] this term only |
| 5 | MeSH descriptor: [Anemia, Pernicious] this term only |
| 6 | "pernicious anemia" or ("vitamin B12" near/3 deficien*):ti,ab,kw |
| 7 | #1 or #2 or #3 or #4 or #5 or #6 |
| 8 | MeSH descriptor: [Prevalence] this term only |
| 9 | prevalen*:ti |
| 10 | MeSH descriptor: [Geography] this term only |
| 11 | MeSH descriptor: [Geography, Medical] this term only |
| 12 | "global health":ti,ab,kw |
| 13 | MeSH descriptor: [World Health] this term only |
| 14 | "world health":ti |
| 15 | MeSH descriptor: [World Health Organization] this term only |
| 16 | (national and survey*) or (population and prevalence):ti,ab,kw |
| 17 | MeSH descriptor: [Geographic Locations] explode all trees |
| 18 | MeSH descriptor: [Population Groups] explode all trees |
| 19 | population:ti |
| 20 | MeSH descriptor: [Epidemiological Monitoring] this term only |
| 21 | MeSH descriptor: [Health Status Indicators] this term only |
| 22 | MeSH descriptor: [Health Surveys] this term only |
| 23 | MeSH descriptor: [Mass Screening] this term only |
| 24 | MeSH descriptor: [Nutrition Assessment] this term only |
| 25 | MeSH descriptor: [Nutrition Surveys] this term only |
| 26 | MeSH descriptor: [Population Surveillance] explode all trees |
| 27 | MeSH descriptor: [Program Evaluation] explode all trees |
| 28 | (program or programs) near/5 evaluat*:ti,ab,kw |
| 29 | #8 or #9 or #10 or #11 or #12 or #13 or #14 or #15 or #16 or #17 or #18 or #19 or #20 or #21 or #22 or #23 or #24 or #25 or #26 or #27 or #28 |
| 30 | #7 and #29 limit 2000-2017 |
