# Supplementary material for: Global folate status in women of reproductive age: a systematic review with emphasis on methodological issues
Source: Ann N Y Acad Sci. 2018 Sep 21;1431(1):35–57. doi: 10.1111/nyas.13963 (PMC6282622; doi:10.1111/nyas.13963)
Supplement: Supplementary file 2 — Appendix S2. Assessment of possible bias in folate concentrations and prevalence estimates due to the choice of assay and cutoff(s). [file NYAS-1431-35-s006.docx]

**Supporting information**

**Appendix S2. Assessment of possible bias in folate concentrations and prevalence estimates due to the choice of assay and cut-off(s)**

Different assays were used in these surveys for the measurement of serum, plasma and/or RBC folate. The three main assay types were the microbiologic assay (MBA), protein-binding assay (PBA), and high-performance liquid chromatography coupled to tandem mass spectrometry (HPLC-MS/MS). Each assay type displayed variations. For the MBA, we specified if it was a traditional (use of wild-type microorganism; MBA_T_) or contemporary (use of chloramphenicol-resistant strain; MBA_C_) assay, and we specified the type of calibrator used (folic acid or 5-methyltetrahydrofolate [5-methyl-THF]). For the PBA, we specified whether the detection was radiometric (i.e., radioimmunoassay [RIA]) or non-radiometric (i.e., immunoassay [IA]). The detection principle of most folate immunoassays is chemiluminescence, but some are based on electro-chemiluminescence or fluorescence polarization. However, knowing the detection principle does not help to interpret assay bias and was therefore not specified. We specified the manufacturer or instrument platform for PBA-type assays (e.g., Roche E170), because this information assists in the interpretation of possible assay bias.

As noted, assays that measure folate status show poor comparability among and sometimes even within the same assay type, posing challenges in comparing data across surveys and over time. To allow better comparisons across surveys, we used data from two proficiency testing programs to compare the performance of the assay used in any given survey with the results of a single comparison assay, the CDC MBA_C_ (using 5-methylTHF as calibrator). This allowed us to estimate an “assay factor” (reported in Tables 2-5), calculated as the ratio of the survey assay results to the CDC MBA_C_ results. We considered the survey assay to measure similar to the CDC MBA_C_ if 0.85 < “assay factor” <1.15, to measure higher than the CDC MBA_C_ if “assay factor” ≥1.15, and to measure lower than the CDC MBA_C_ if “assay factor” ≤0.85. Additionally, because prevalence estimates for folate deficiency and insufficiency may be biased if they are based on a cut-off derived using a different assay, we also estimated a “cut-off factor” (reported in Tables 2-5), calculated as the ratio of the survey cut-off to the recommended MBA_C_ cut-off (e.g., <7 nmol/L for serum deficiency, <305 nmol/L for RBC deficiency, or <748 nmol/L for RBC insufficiency). Lastly, we estimated a “prevalence factor” (reported in Tables 2-5) which is the combination of the other two factors and was calculated by dividing the “assay factor” by the “cut-off factor”. By having a general understanding of how the various assays performed relative to the MBA_C_, and by considering the cut-off values used in each survey for defining folate deficiency or insufficiency, we could better assess whether the reported prevalence estimates were likely correct (0.85 < “prevalence factor” <1.15) or may represent an under- (“prevalence factor” ≥1.15) or overestimation (“prevalence factor” ≤0.85).

As noted above, data from two proficiency testing programs was used for assessing possible assay bias. The College of American Pathologists (CAP) Ligand Survey (<http://www.cap.org>) is a large proficiency testing program (about 1,000 participating laboratories) that provides refrigerated serum (n=3) or whole blood samples (n=2) to mainly US-based laboratories three times a year. The United Kingdom National External Quality Assessment (UK NEQAS) Haematinics Survey (<https://ukneqas.org.uk/>) is a medium proficiency testing program (about 200 participating laboratories) that provides refrigerated serum (n=3) or whole blood samples (n=2) to mainly UK-based laboratories 11 times a year. Because the UK NEQAS program has a much larger number of data points (33 and 22 per year for serum and whole blood, respectively) compared to the CAP program (9 and 6 per year for serum and whole blood, respectively), we used information from the UK NEQAS program when possible. However, the CDC MBA_C_ participated in the UK NEQAS program only since 2009. The CDC participated in the CAP program up to 2006 using the Bio-Rad RIA, when the manufacturer discontinued the assay. Thus, prior to 2009, a survey assay’s performance could be compared to the Bio-Rad RIA, which could then be compared to the CDC MBA_C_ because the CDC has well-documented information on how these two assays relate to each other.^19^ Whenever possible, we utilized proficiency testing data generated as close as possible in time to when the survey testing was performed. Unfortunately, after 2002, too few laboratories using the Bio-Rad RIA or other commercial RIA assays participated in the CAP program, and so the CAP report no longer provided information for these assays. Therefore, for surveys conducted after 2002 that used an RIA assay type, we used the 2002 CAP data as a reference. For surveys that used an MBA_C_ assay, we used previously reported information on how results compare when the assay is calibrated with folic acid vs. 5-methylTHF.^20^

We illustrate the process of estimating the “assay factor”, “cutoff factor”, and “prevalence factor” using two examples. First, the 7^th^ National Nutrition Survey in the Philippines reported the prevalence of serum folate <7 nmol/L and RBC folate <397 nmol/L as assessed using the DPC Dual Count™ RIA (Table 3). For serum folate, this assay measured 65% higher than the Bio-Rad RIA and the Bio-Rad RIA measured 30% lower than the CDC MBA_C_; thus, the “assay factor” was 1.65 * 0.70 = 1.16; in other words, the DPC Dual Count™ RIA measured 16% higher than the CDC MBA_C_ (Supplemental Table S1, Supporting Information; online only). Because the “cut-off factor” for serum folate was 1 (survey cut-off was the same as the MBA_C_ cut-off for deficiency), the “prevalence factor” was 1.16. Because the “prevalence factor” was more than 15% higher than 1, we therefore concluded that the reported prevalence of serum folate deficiency likely underestimated deficiency. However, because we had no proficiency testing data for RBC folate for that assay, we could not assess possible bias in RBC folate deficiency. Second, the 2014 Cambodian Micronutrient survey reported the prevalence of serum folate <10 nmol/L as assessed using the Roche cobas® e411 IA (Table 2). For serum folate, this assay measured 59% higher than the CDC MBA_C_ (Supplemental Table S1, Supporting Information; online only); thus, the “assay factor” was 1.59 (Table 2). The “cut-off factor” was 1.43 (10/7) and the “prevalence factor” was 1.11 (1.59/1.43). Because the “prevalence factor” deviated by <15% from 1, we concluded that the reported prevalence of serum folate deficiency was likely correct. These two examples show how important it is to consider both how the survey assay measured relative to the CDC MBA_C_ and how the survey cut-off compared to the recommended MBA_C_ cut-off, when interpreting prevalence estimates.

In an attempt to simplify the cut-off values presented in this article, we rounded to the nearest integer (e.g., <7 nmol/L instead of <6.8 nmol/L).
