# Supplementary material for: Global folate status in women of reproductive age: a systematic review with emphasis on methodological issues
Source: Ann N Y Acad Sci. 2018 Sep 21;1431(1):35–57. doi: 10.1111/nyas.13963 (PMC6282622; doi:10.1111/nyas.13963)
Supplement: Supplementary file 3 — Table S1. Data on assay performance and specimen handling and storage to inform the interpretation of results. [file NYAS-1431-35-s004.docx]

**Supplementary Table S1.** Data on assay performance and specimen handling and storage to inform interpretation of survey results.

| Country | Year | Matrix | Survey assay | Laboratory | Proficiency testing data used to assess assay performance | Comment about assay performance | Comment about specimen handling and storage |
| --- | --- | --- | --- | --- | --- | --- | --- |
| **Countries with low-income economies** | | | | | | | |
| Afghanistan | 2013 | Serum | Not reported | Pakistan, Nutrition Research Laboratory, Aga Khan University | N/A | Results cannot be interpreted without knowing survey assay | Cold-chain was maintained, but no information on how long specimens were stored or at what temperature |
| Cambodia | 2014 | Plasma | PBA (IA; Roche cobas® e411) | Cambodia, Institut Pasteur | UK NEQAS; n = 27 (2014) | Measured on average 59% (range: 6% to 153%) higher than CDC MBA_C_ with 5-methyl-THF calibrator; concentration-dependent bias decreased from low to high concentration | Cold-chain was maintained, but no information on how long specimens were stored or at what temperature |
| Ethiopia | 2005 | Serum | PBA (IA; Roche Elecsys®) | Ethiopia, Ethiopian Health and Nutrition Research Institute | CAP; n = 9 (2002) | Measured on average 64% (range: -3% to 140%) higher than Bio-Rad RIA, which corresponds to 15% higher than the CDC MBA_C_ with 5-methyl-THF calibrator; concentration-dependent bias decreased from low to high concentration | Specimen handling and storage seems appropriate |
| Sierra Leone | 2013 | Plasma | PBA (IA; Roche cobas® e411) | the United States, ARS-Western Human Nutrition Research Center/UC Davis | UK NEQAS; n = 27 (2014) | Measured on average 59% (range: 6% to 153%) higher than CDC MBA_C_ with 5-methyl-THF calibrator; concentration-dependent bias decreased from low to high concentration | Specimen handling and storage seems appropriate; however, samples heated to 60 ˚C for 60 min prior to analysis due to concerns about Ebola virus; same heating procedure applied to 32 samples with known values showed only negligible folate deterioration |
| **Countries with lower-middle-income economies** | | | | | | | |
| Bangladesh | 2011–2012 | Serum | PBA (IA; Roche cobas® e601) | Bangladesh, International Centre for Diarrhoeal Disease Research | UK NEQAS; n = 33 (2012) | Measured on average 63% (range: 13% to 139%) higher than CDC MBA_C_ with 5-methyl-THF calibrator; concentration-dependent bias decreased from low to high concentration | Specimen handling and storage seems appropriate |
| Cameroon | 2009 | Plasma | PBA (RIA; MP Biomedicals SimulTRAC-SNB) | the United States, ARS-Western Human Nutrition Research Center/UC Davis | CAP; n = 9 (2002) | Measured on average 92% (range: 33% to 126%) higher than Bio-Rad RIA, which corresponds to 34% higher than the CDC MBA_C_ with 5-methyl-THF calibrator; no proficiency testing data available for 2009 | Specimen handling and storage seems appropriate |
| Cote d'Ivoire | 2007 | Plasma | MBA_C_ (folic acid calibrator) | Switzerland, Swiss Vitamin Institute | No proficiency testing data available | Assume that MBA_C_ calibrated with folic acid measured 20% higher than CDC MBA_C_ with 5-methyl-THF calibrator | Specimen handling seems appropriate, but specimen storage at -25°C for ~9 months may have caused folate losses |
| Georgia | 2009 | Plasma | MBA_C_ (folic acid calibrator; DRG International) | Georgia, Imereti Zonal Diagnostic Laboratory, Georgia National Center for Disease Control and Public Health | No proficiency testing data available | Assume that MBA_C_ calibrated with folic acid measured 20% higher than CDC MBA_C_ with 5-methyl-THF calibrator | Specimen handling and storage seems appropriate |
| Guatemala | 2009–2010 | Serum, RBC | MBA_C_ (5-methyl-THF calibrator) | the United States, Centers for Disease Control and Prevention | N/A | Survey assay is CDC MBA_C_ with 5-methyl-THF calibrator | Specimen handling and storage seems appropriate |
| Kyrgyzstan | 2009 | Dried blood spots | MBA_C_ (folic acid calibrator) | Ireland, Hematology Laboratory, St. James Hospital Dublin | No proficiency testing data available | QC sample exchange with CDC MBA_C_ with folic acid calibrator showed similar results; assume that MBA_C_ calibrated with folic acid measured 20% higher than CDC MBA_C_ with 5-methyl-THF calibrator | Specimen handling and storage seems appropriate |
| Philippines | 2008 | Serum, RBC | PBA (RIA; DPC Dual Count™ Solid Phase No Boil) | Philippines, Food and Nutrition Research Institute, Department of Science and Technology | CAP; n = 9 for serum (2002) | Serum: measured on average 65% (range: 26% to 156%) higher than Bio-Rad RIA, which corresponds to 16% higher than the CDC MBA_C_ with 5-methyl-THF calibrator; no proficiency testing data available for RBC or for either matrix for 2008 | Specimen handling and storage seems appropriate |
| Uzbekistan | 2008 | Serum | MBA_C_ (5-methyl-THF calibrator) | Uzbekistan, Department of Hematology, Tashkent Hematology and Blood Transfusion Center | No proficiency testing data available | QC sample exchange with CDC MBA_C_ with 5-methyl-THF calibrator showed similar results | Specimen handling and storage seems appropriate; the 2-4 day delay in freezing serum is not ideal, but should not have led to more than 5-10% folate losses |
| Viet Nam | 2010 | Plasma | MBA_C_ (folic acid calibrator) | Switzerland, Swiss Vitamin Institute | No proficiency testing data available | Assume that MBA_C_ calibrated with folic acid measured 20% higher than CDC MBA_C_ with 5-methyl-THF calibrator | Specimen handling and storage seems appropriate |
| **Countries with upper-middle-income economies** | | | | | | | |
| Azerbaijan | 2013 | Plasma | MBA_C_ (5-methyl-THF calibrator) | Switzerland, Swiss Vitamin Institute | No proficiency testing data available | Assume that MBA_C_ calibrated with 5-methylTHF measured similar to CDC MBA_C_ with 5-methyl-THF calibrator | Specimen handling and storage seems appropriate |
| Belize | 2011 | Serum, RBC | MBA_C_ (5-methyl-THF calibrator) | the United States, University of Florida | N/A | Survey assay harmonized^a^ to CDC MBA_C_ with 5-methyl-THF calibrator | Specimen handling and storage seems appropriate |
| China, Shaanxi | 2008 | Plasma | PBA (RIA; MP Biomedicals SimulTRAC-SNB) | China, Xi'an Jiaotong University | CAP; n = 9 (2002) | Measured on average 92% (range: 33% to 126%) higher than Bio-Rad RIA, which corresponds to 34% higher than the CDC MBA_C_ with 5-methyl-THF calibrator; no proficiency testing data available for 2008 | Specimen handling and storage seems appropriate |
| China, Taiwan | 2005–2008 | Serum | PBA (IA; DPC IMMULITE® 2000) | Not reported | CAP; n = 9 (2002) | Measured on average 59% (range: 30% to 103%) higher than Bio-Rad RIA, which corresponds to 11% higher than the CDC MBA_C_ with 5-methyl-THF calibrator; no proficiency testing data available for 2005-2008 | Specimen handling and storage seems appropriate |
| Dominican Republic | 2009 | Serum, RBC | MBA_C_ (folic acid calibrator) | Peru, Instituto de Investigaciones Nutricionales | No proficiency testing data available | Assume that MBA_C_ calibrated with folic acid measured 20% higher than CDC MBA_C_ with 5-methyl-THF calibrator | Specimen handling and storage seems appropriate |
| Ecuador | 2012 | Serum, RBC | PBA (IA; Siemens IMMULITE® 2000) | Ecuador, Netlab S.A. Laboratorios Especializados de Referencia | UK NEQAS; n = 33 for serum and n = 18 for RBC (2012) | Serum: measured on average 3% (range: -27% to 57%) higher than CDC MBA_C_ with 5-methyl-THF calibrator; RBC: measured on average 62% (range: 14% to 210%) higher than CDC MBA_C_ with 5-methyl-THF calibrator | Specimen handling seems appropriate; no information on specimen storage temperature |
| Fiji | 2004 | Serum | PBA (IA; Roche E170) | New Zealand, Capital & Coast District Health Board Laboratory | CAP; n = 9 (2002) | Measured on average 64% (range: -3% to 140%) higher than Bio-Rad RIA, which corresponds to 15% higher than the CDC MBA_C_ with 5-methyl-THF calibrator | Specimen handling and storage seems appropriate |
| Fiji | 2010 | Serum | PBA (IA; Roche E170) | New Zealand, TropicHealth Laboratories | UK NEQAS; n = 32 (2010) | Measured on average 42% (range: 1% to 103%) higher than CDC MBA_C_ with 5-methyl-THF calibrator; concentration-dependent bias decreased from low to high concentration | Specimen handling and storage seems appropriate |
| Iran (the Islamic Republic of) | 2006 | Serum | PBA (RIA; MP Biomedicals SimulTRAC-SNB) | Iran (the Islamic Republic of), National Reference Laboratory | CAP; n = 9 (2002) | Measured on average 92% (range: 33% to 126%) higher than Bio-Rad RIA, which corresponds to 34% higher than the CDC MBA_C_ with 5-methyl-THF calibrator; no proficiency testing data available for 2006 | Specimen handling and storage seems appropriate |
| Iran (the Islamic Republic of) | 2008 | Serum | PBA (RIA; MP Biomedicals SimulTRAC-SNB) | Iran (the Islamic Republic of), National Reference Laboratory | CAP; n = 9 (2002) | Measured on average 92% (range: 33% to 126%) higher than Bio-Rad RIA, which corresponds to 34% higher than the CDC MBA_C_ with 5-methyl-THF calibrator; no proficiency testing data available for 2008 | Specimen handling and storage seems appropriate |
| Iraq | 2011–2012 | RBC | MBA_C_ (5-methyl-THF calibrator) | Jordan, Central Public Health Laboratory | No proficiency testing data available | Survey assay harmonized^a^ to CDC MBA_C_ with 5-methyl-THF calibrator | Specimen handling and storage seems appropriate; RBC folate concentrations were not corrected for the contribution of serum folate, which may result in slightly higher RBC folate concentrations |
| Jordan | 2010 | RBC | MBA_C_ (folic acid calibrator) | Ireland, Hematology Laboratory, St. James Hospital Dublin | No proficiency testing data available | QC sample exchange with CDC MBA_C_ with folic acid calibrator showed similar results; assume that MBA_C_ calibrated with folic acid measured 20% higher than CDC MBA_C_ with 5-methyl-THF calibrator | Cold-chain was maintained, but no information on how long specimens were stored or at what temperature |
| Lebanon | 2003 | Plasma | PBA (IA; Abbott AXSYM®) | Lebanon, American University of Beirut | CAP; n = 9 (2002) | Measured on average 94% (range: 52% to 230%) higher than Bio-Rad RIA, which corresponds to 36% higher than the CDC MBA_C_ with 5-methyl-THF calibrator | Specimen handling seems appropriate; no information on specimen storage temperature |
| Mexico | 2012 | Serum | PBA (IA; Abbott Architect^TM^) | Mexico, National Institute of Public Health | UK NEQAS; n = 33 (2011) | Measured on average 14% (range: -11% to 48%) higher than CDC MBA_C_ with 5-methyl-THF calibrator | Specimen handling and storage seems appropriate |
| Mongolia  (Regional) | 2001 | Serum | PBA (RIA: Bio-Rad Quantaphase® II) | the United States, Centers for Disease Control and Prevention | N/A | Survey assay measured 30% lower than CDC MBA_C_ with 5-methyl-THF calibrator^17^ | Specimen handling and storage seems appropriate |
| Mongolia | 2004 | Serum | PBA (RIA: assay not specified) | Mongolia, Nuclear Diagnostic Department, Clinical Hospital No 1 | N/A | Results cannot be interpreted without knowing survey assay | Cold-chain was maintained, but no information on how long specimens were stored or at what temperature |
| South Africa | 2005 | Serum, RBC | PBA (IA Beckman Coulter® Access® and Bayer ADVIA Centaur®) | South Africa, PathCare and Lancet Laboratories | CAP; n = 9 for serum and n = 6 for RBC (2002) | Serum: Beckman assay measured on average 34% (range: 7% to 48%) and Bayer assay measured on average 43% (range: 24% to 82%) higher than Bio-Rad RIA, which corresponds to 6% lower and comparable (0% difference) to the CDC MBA_C_ with 5-methyl-THF calibrator, respectively; RBC: Beckman assay measured on average 155% (range: 118% to 159%) and Bayer assay measured on average 46% (range: -11% to 130%) higher than Bio-Rad RIA, which corresponds to 79% and 2% higher than the CDC MBA_C_ with 5-methyl-THF calibrator, respectively; no proficiency testing data available for 2005 | Specimen handling and storage seems appropriate |
| Turkey Edirne | Unknown; before 2006 | Serum | PBA (IA; DPC IMMULITE® 2000) | Turkey, Nuclear Medicine Laboratory | CAP; n = 9 (2002) | Measured on average 59% (range: 30% to 103%) higher than Bio-Rad RIA, which corresponds to 11% higher than the CDC MBA_C_ with 5-methyl-THF calibrator; no proficiency testing data available for around 2006 | Specimen handling and storage seems appropriate |
| **Countries with high-income economies** | | | | | | | |
| Argentina | 2004–2005 | Serum | PBA (IA; Roche E170) | Argentina, Centro de Estudios Infectologicos S.A. | CAP; n = 9 (2002) | Measured on average 64% (range: -3% to 140%) higher than Bio-Rad RIA, which corresponds to 15% higher than the CDC MBA_C_ with 5-methyl-THF calibrator | Specimen handling and storage seems appropriate |
| Australia | 2011–2012 | Serum, RBC | PBA (IA; Roche E170) | Australia, Douglass Hanly Moir Pathology Laboratory | UK NEQAS; n = 32 for serum and n = 22 for RBC (2010) | Serum: measured on average 42% (range: 1% to 103%) higher than CDC MBA_C_ with 5-methyl-THF calibrator; concentration-dependent bias decreased from low to high concentration; RBC: measured on average 282% (range: 100% to 536%) higher than CDC MBA_C_ with 5-methyl-THF calibrator | No information on cold-chain, but turnaround time from collection to analysis was less than 72 hours |
| Austria | 2010–2012 | Plasma | PBA (RIA; MP Biomedicals SimulTRAC-SNB) | Not reported | CAP; n = 9 (2002) | Measured on average 92% (range: 33% to 126%) higher than Bio-Rad RIA, which corresponds to 34% higher than the CDC MBA_C_ with 5-methyl-THF calibrator; no proficiency testing data available for 2010-2012 | Specimen handling and storage seems appropriate |
| Bahrain | 2002 | Serum | Not reported | Bahrain, Salmaniya Medical Complex | N/A | Results cannot be interpreted without knowing survey assay | Specimen handling and storage seems appropriate |
| Canada  Newfoundland | 2000–2001 | Serum, RBC | Not reported | Canada, Health Care Corporation | N/A | Results cannot be interpreted without knowing survey assay | No information provided |
| Canada | 2007-2009 | RBC | PBA (IA; Siemens IMMULITE® 2000) | Canada, Health Canada Nutrition Laboratory | UK NEQAS; n = 22 (2009) | Measured on average 70% (range: 8% to 178%) higher than CDC MBA_C_ with 5-methyl-THF calibrator | Specimen handling and storage seems appropriate |
| France | 2006-2007 | Plasma | PBA (IA; manufacturer not specified) | Not reported | N/A | Results cannot be interpreted without knowing survey assay | Specimen handling and storage seems appropriate |
| Ireland | 2008–2010 | Serum, RBC | MBA_C_ (folic acid calibrator) | Ireland, Trinity College Dublin | N/A | Measured ~20% higher than CDC MBA_C_ with 5-methyl-THF calibrator^18^ | Specimen handling and storage seems appropriate |
| New Zealand | 2008–2009 | Serum, RBC | MBA_C_ (folic acid calibrator) | New Zealand, University of Otago | No proficiency testing data available | Assume that MBA_C_ calibrated with folic acid measured 20% higher than CDC MBA_C_ with 5-methyl-THF calibrator | Specimen handling and storage seems appropriate |
| Spain  Madrid region | Unknown; before 2009 | Serum | PBA (IA; Roche E170) | Not reported | UK NEQAS; n = 32 (2010) | Measured on average 42% (range: 1% to 103%) higher than CDC MBA_C_ with 5-methyl-THF calibrator; concentration-dependent bias decreased from low to high concentration | Specimen handling and storage seems appropriate |
| Sweden | 2010–2011 | Plasma, RBC | PBA (IA; Abbott Architect^TM^) | Sweden, Karolinska University Hospital (RBC) and Uppsala University Hospital (plasma) | UK NEQAS; n = 33 (2011) | Serum: measured on average 14% (range: -11% to 48%) higher than CDC MBA_C_ with 5-methyl-THF calibrator; RBC: measured on average 27% (range: -9% to 63%) higher than CDC MBA_C_ with 5-methyl-THF calibrator | Specimen handling seems appropriate; no information on how long specimens were stored at -20°C |
| the United Kingdom | 2000–2001 | Serum, RBC | PBA (IA; Abbott IMx^TM^) | the United Kingdom, Department of Haematology, Great Ormond Street Hospital | No proficiency testing data available | Results cannot be interpreted | Specimen handling and storage seems appropriate except for shipment of freshly collected blood by post to laboratory, which may have led to small folate losses |
| the United Kingdom | 2008–2012 | Serum, RBC | HPLC-MS/MS for serum; MBA_C_ (5-methyl-THF calibrator) for RBC | the United States, Centers for Disease Control and Prevention | N/A | Survey assay is CDC MBA_C_ with 5-methyl-THF calibrator; CDC HPLC-MS/MS for serum is comparable to CDC MBA_C_ with 5-methyl-THF calibrator^84^ | Specimen handling and storage seems appropriate except for shipment of freshly collected blood by post to laboratory, which may have led to small folate losses |
| the United States | 2007–2012 | Serum, RBC | MBA_C_ (5-methyl-THF calibrator); HPLC-MS/MS for serum during 2011-2012 | the United States, Centers for Disease Control and Prevention | N/A | Survey assay is CDC MBA_C_ with 5-methyl-THF calibrator; CDC HPLC-MS/MS for serum is comparable to CDC MBA_C_ with 5-methyl-THF calibrator^84^ | Specimen handling and storage seems appropriate |

^a^ Laboratory adapted same assay procedure as CDC MBA_C_

Abbreviations: 5-methyl-THF, 5-methyltetrahydrofolate; CAP, College of American Pathologists Ligand Survey; CDC, US Centers for Disease Control and Prevention; MBA, microbiologic assay; MBA_C_, contemporary MBA; IA, immunoassay; N/A, not applicable; PBA, protein-binding assay; QC, quality control; RBC, red blood cell; RIA, radioimmunoassay; UC-Davis, University of California-Davis; UK NEQAS, United Kingdom National External Quality Assessment.

Disclaimer: The mention of specific companies or of certain manufacturers’ products does not imply that they are endorsed or recommended by the World Health Organization in preference to others of a similar nature that are not mentioned. Errors and omissions excepted, the names of proprietary products are distinguished by initial capital letters.
