# Supplementary figures and images for: Global folate status in women of reproductive age: a systematic review with emphasis on methodological issues
Source: Ann N Y Acad Sci. 2018 Sep 21;1431(1):35–57. doi: 10.1111/nyas.13963 (PMC6282622; doi:10.1111/nyas.13963)

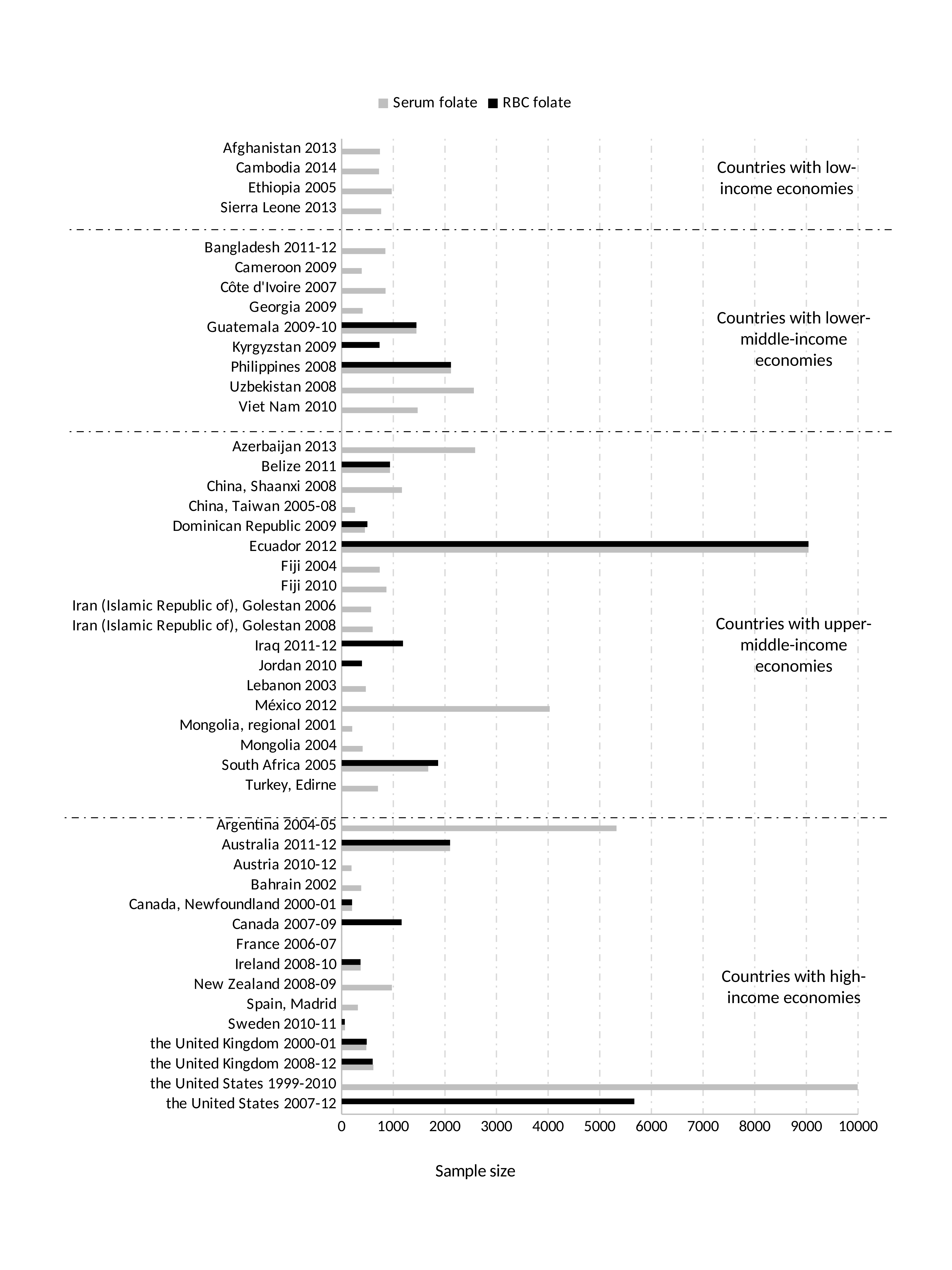

Supplement: Supplementary file 4 — Figure S1. Surveys included in this article grouped by income category and indicating sample sizes available for serum and RBC folate. Gray bars indicate serum/plasma folate and black bars indicate RBC folate. [file NYAS-1431-35-s001.tiff]

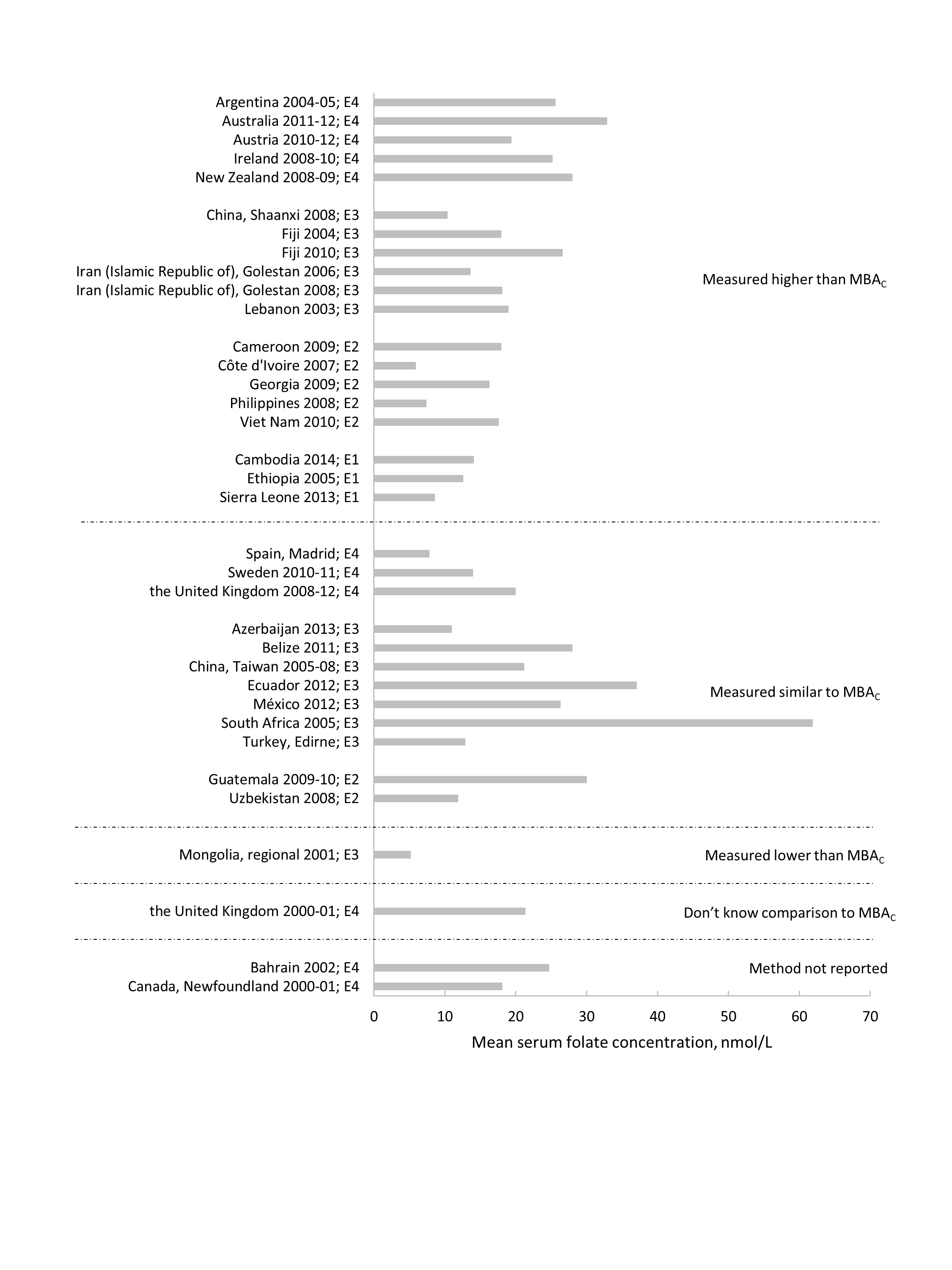

Supplement: Supplementary file 5 — Figure S2. Reported mean serum/plasma folate concentrations in women of reproductive age by the survey, indicating an interpretation of the data based on the assay used in the survey relative to the CDC MBAC. [file NYAS-1431-35-s002.tif]

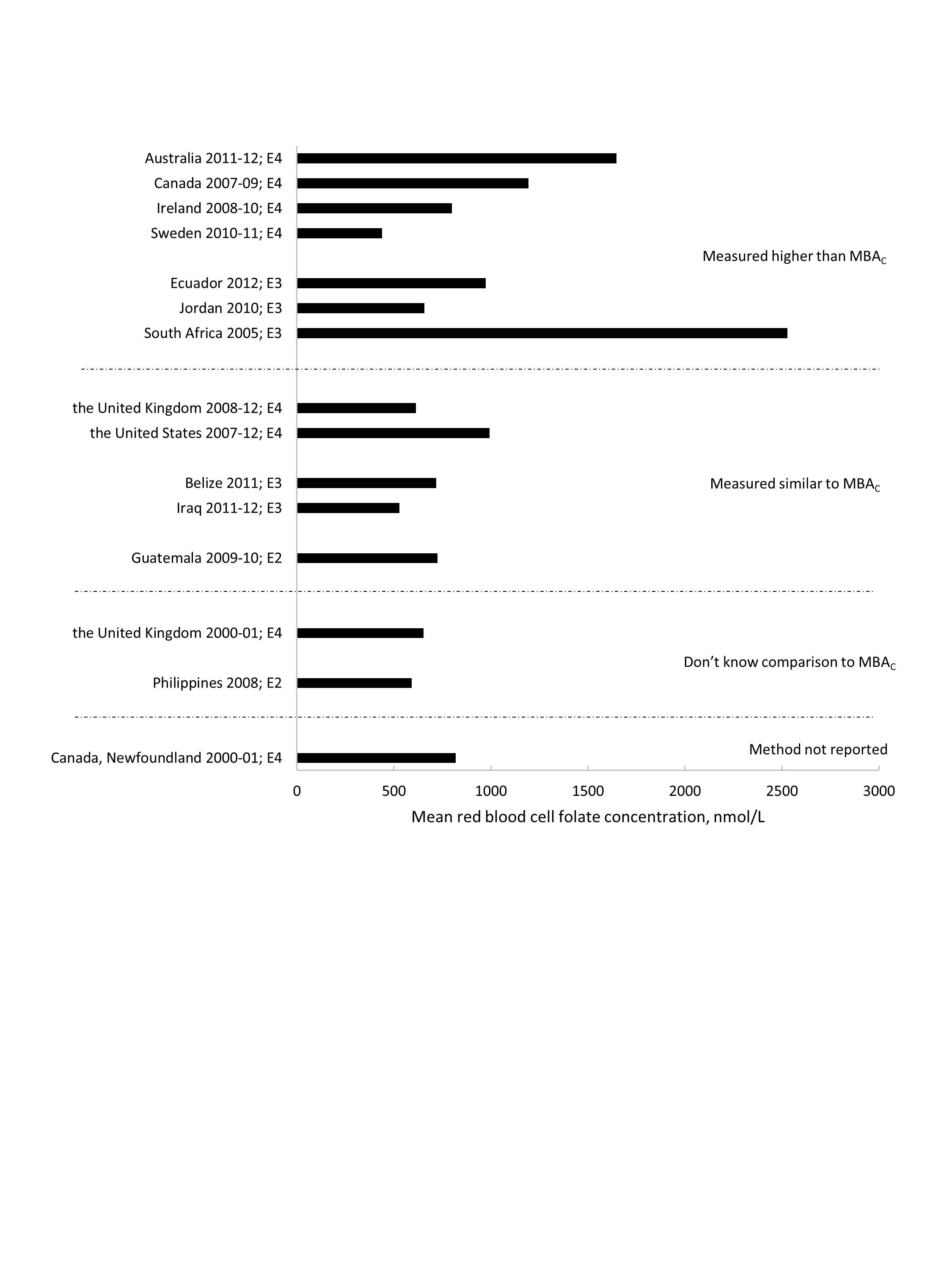

Supplement: Supplementary file 6 — Figure S3. Reported mean red blood cell folate concentrations in women of reproductive age by the survey, indicating an interpretation of the data based on the assay used in the survey relative to the CDC MBAC. [file NYAS-1431-35-s003.tif]
