# Supplementary material for: Global folate status in women of reproductive age: a systematic review with emphasis on methodological issues
Source: Ann N Y Acad Sci. 2018 Sep 21;1431(1):35–57. doi: 10.1111/nyas.13963 (PMC6282622; doi:10.1111/nyas.13963)
Supplement: Supplementary file 7 [file NYAS-1431-35-s007.docx]

**Supplementary figure captions:**

**Figure S1**. Surveys included in this article grouped by income category and indicating sample sizes available for serum and RBC folate. Grey bars indicate serum/plasma folate and black bars indicate RBC folate.

**Figure S2****.** Reported mean serum/plasma folate concentrations in women of reproductive age by survey, indicating an interpretation of the data based on the assay used in the survey relative to the CDC MBA_C_. Data from one of two proficiency-testing programs were used to compare the performance of the survey assay with the performance of the CDC MBA_C_ calibrated with 5-methyl-THF as the comparison assay. In the proficiency testing programs, each participating laboratory applies its assay to the same panel of samples, allowing us to estimate an “assay factor,” or the extent to which one assay measures higher or lower than another, by calculating the ratio of the survey assay’s results to the CDC MBA_C_. Abbreviations: CDC, US Centers for Disease Control and Prevention; MBA_C_, contemporary microbiologic assay. The abbreviation after each survey indicates the economy: E1, low income; E2 lower-middle income; E3, upper-middle income; E4, high income.

**Figure S3.** Reported mean red blood cell folate concentrations in women of reproductive age by survey, indicating an interpretation of the data based on the assay used in the survey relative to the CDC MBA_C_. Data from one of two proficiency-testing programs were used to compare the performance of the survey assay with the performance of the CDC MBA_C_ calibrated with 5-methyl-THF as the comparison assay. In the proficiency testing programs, each participating laboratory applies its assay to the same panel of samples, allowing us to estimate an “assay factor,” or the extent to which one assay measures higher or lower than another, by calculating the ratio of the survey assay’s results to the CDC MBA_C_. Abbreviations: CDC, US Centers for Disease Control and Prevention; MBA_C_, contemporary microbiologic assay. The abbreviation after each survey indicates the economy: E1, low income; E2 lower-middle income; E3, upper-middle income; E4, high income.
